# Brazilian Society of Otology task force – Vestibular Schwannoma ‒ evaluation and treatment^☆^

**DOI:** 10.1016/j.bjorl.2023.101313

**Published:** 2023-08-28

**Authors:** Vagner Antonio Rodrigues Silva, Joel Lavinsky, Henrique Furlan Pauna, Melissa Ferreira Vianna, Vanessa Mazanek Santos, Cláudio Márcio Yudi Ikino, André Luiz Lopes Sampaio, Paula Tardim Lopes, Pauliana Lamounier, André Souza de Albuquerque Maranhão, Vitor Yamashiro Rocha Soares, José Fernando Polanski, Mariana Moreira de Castro Denaro, Carlos Takahiro Chone, Ricardo Ferreira Bento, Arthur Menino Castilho

**Affiliations:** a Universidade Estadual de Campinas (Unicamp), Faculdade de Ciências Médicas (FCM), Departamento de Otorrinolaringologia e Cirurgia de Cabeça e Pescoço, Campinas, SP, Brazil; b Sociedade Brasileira de Otologia - SBO; c Universidade Federal do Rio Grande do Sul (UFRGS), Departamento de Ciências Morfológicas, Porto Alegre, RS, Brazil; d Hospital Universitário Cajuru, Departamento de Otorrinolaringologia, Curitiba, PR, Brazil; e Irmandade Santa Casa de Misericórdia de São Paulo, Departamento de Otorrinolaringologia, São Paulo, SP, Brazil; f Universidade Federal do Paraná, Hospital de Clínicas, Departamento de Otorrinolaringologia e Cirurgia de Cabeça e Pescoço, Curitiba, PR, Brazil; g Universidade Federal de Santa Catarina, Hospital Universitário, Departamento de Cirurgia, Florianópolis, SC, Brazil; h Universidade de Brasília (UnB), Faculdade de Medicina, Laboratório de Ensino e Pesquisa em Otorrinolaringologia, Brasília, DF, Brazil; i Faculdade de Medicina da Universidade de São Paulo (FMUSP), Departamento de Otorrinolaringologia, São Paulo, SP, Brazil; j Centro de Reabilitação e Readaptação Dr. Henrique Santillo (CRER), Departamento de Otorrinolaringologia, Goiânia, GO, Brazil; k Universidade Federal de São Paulo (UNIFESP), Escola Paulista de Medicina, Departamento de Otorrinolaringologia e Cirurgia de Cabeça e Pescoço, São Paulo, SP, Brazil; l Hospital Flavio Santos e Hospital Getúlio Vargas, Grupo de Otologia e Base Lateral do Crânio, Teresina, PI, Brazil; m Faculdade Evangélica Mackenzie do Paraná, Faculdade de Medicina, Curitiba, PR, Brazil; n Universidade Federal de Minas Gerais (UFMG), Hospital das Clínicas, Belo Horizonte, MG, Brazil

**Keywords:** Vestibular schwannoma, Neuroma, acoustic, Hearing loss, sensorineural, Single sided deafness, Radiotherapy, Neurofibromatosis 2, Auditory brain stem implants, Cochlear implant, Surgery

## Abstract

**Objective:**

To review the literature on the diagnosis and treatment of vestibular schwannoma. Methods: Task force members were educated on knowledge synthesis methods, including electronic database search, review and selection of relevant citations, and critical appraisal of selected studies. Articles written in English or Portuguese on vestibular schwannoma were eligible for inclusion. The American College of Physicians’ guideline grading system and the American Thyroid Association’s guideline criteria were used for critical appraisal of evidence and recommendations for therapeutic interventions.

**Results:**

The topics were divided into 2 parts: (1) Diagnosis — audiologic, electrophysiologic tests, and imaging; (2) Treatment — wait and scan protocols, surgery, radiosurgery/radiotherapy, and systemic therapy.

**Conclusions:**

Decision making in VS treatment has become more challenging. MRI can diagnose increasingly smaller tumors, which has disastrous consequences for the patients and their families. It is important to develop an individualized approach for each case, which highly depends on the experience of each surgical team.

## Highlights

• In asymmetric SNHL, MRI should be performed regardless of ABR results.
• Gadolinium-enhanced MRI is the gold standard exam for suspected VS.
• Intervention is indicated for tumors with significant growth (≥2 mm/year) and/or on Koos grade III and IV.
• Radiotherapy may be indicated as primary treatment in selected cases; however, there is still controversy surrounding long-term effectiveness and safety, especially in young patients.
• Surgery is superior to radiotherapy in order to preserve serviceable hearing in patients with VS.

## Introduction

Vestibular Schwannomas (VSs) are the most common extra-axial tumor of the posterior fossa in adults and account for more than 80% of tumors in the Cerebellopontine Angle (CPA).^1^, ^2^ They are the third most common nonmalignant intracranial tumors after meningiomas and pituitary adenomas.^3^ VSs are typically unilateral, and bilateral VSs are associated with Neurofibromatosis type 2 (NF2). They appear anywhere along Cranial Nerve (CN) VIII,^4^ and the inferior vestibular branch is affected in 85% of cases.^5^

VS diagnosis is made according to the 2021 World Health Organization (WHO) classification.^6^ Most patients present unilateral Sensorineural Hearing Loss (SNHL) (94%) and tinnitus (83%), but the frequency of vestibular symptoms is highly variable and probably underreported.^7^ Large tumors can cause tingling in the face as a result of trigeminal nerve compression, as well as facial paralysis, brainstem compression, and hydrocephalus.

According to the US Central Brain Tumor Registry, the overall incidence of VS between 2004 and 2010 was 1.09 per 100,000 year.^8^ It increases with age, reaching a peak of 2.93 per 100,000 year in the age group of 65–74 years, with no difference related to gender. There is substantial geographic variation in the incidence of VS: a recent analysis of the Surveillance, Epidemiology, and End Results (SEER) database in the USA revealed that the annual incidence of VS is lowest among Black and Hispanic populations and highest among Caucasian ones (*p* < 0.001).^9^ These differences in incidence may be due to genetic and environmental factors, as well as different diagnostic practices. Improved screening protocols for asymmetric hearing loss, better access to images, and higher Magnetic Resonance Imaging (MRI) resolution have led to an increase in diagnosis and a decrease in mean tumor size at diagnosis.^10^

Risk factors for VS have been little investigated. A population-based case-control study conducted in the UK and Nordic countries revealed that childbirth women were at higher risk for VS.^11^ There was no association with age at first birth or number of children. Tumor risk was lower in current smokers but not in former smokers. The use of cell phones has already been associated with the onset of VS. However, no clinical association has been demonstrated between cell and cordless telephone use and VS.^12^ The quality of published studies is limited by their retrospective nature. The biological mechanisms, if any, underlying these associations remain unclear.

### Histopathology

Histologic features of conventional VS in HE stains had specific morphologic features in most cases. These include cellular Antoni A areas composed of interlacing bundles of spindle cells alternating with loose hypocellular and microcystic Antoni B areas, as well as Verocay bodies consisting of arrangements of palisade nuclei alternating with zones containing cellular processes. Immunohistochemically, VSs are diffusely positive for S100B and SOX10.^13^

Cellular and melanotic schwannomas are variants that may raise important considerations in differential diagnosis. Cellular schwannomas are characterized by hypercellularity and a predominance or exclusivity of an Antoni A pattern without Verocay (well-formed) bodies.^14^ These tumors are benign and, therefore, the distinction from Malignant Peripheral Nerve Sheath Tumors (MPNSTs) is important.^15^, ^16^ Melanocytic schwannomas are recognized by the WHO as a distinct entity that rarely affects the CNs.^17^, ^18^ Melanocytic schwannomas are grossly pigmented and express melanocytic markers such as HMB45 and melan-A, leading to a distinct differential diagnosis that includes melanoma. The psammomatous melanocytic schwannoma subvariant has a 50% association with Carney complex, an autosomal dominant clinical condition characterized by myxomas, hyperpigmentation, and endocrine hyperactivity. Unlike conventional or cellular schwannomas, there is a 10% risk of malignant transformation in melanocytic schwannoma.^19^

### Molecular biology

Molecular analyses do not currently play a role in diagnosis, prognosis, or therapeutic guidance. Hotspot mutations in the GNAQ/GNA11, BRAF, and pTERT genes are useful for differentiating melanotic schwannoma (wild type) from melanocytoma (typically a GNAQ/GNA11 mutation) and cutaneous melanoma (typically a BRAF or pTERT mutation).^20^, ^21^ Epigenetic analyses using genome-wide methylation profiles emerge as an excellent tool to differentiate groups of biologically distinct tumors. Most VSs form a methylation cluster that is different from that of schwannomas in other locations. Methylation profiles also differentiate schwannomas (cellular) from histologic mimics.^15^, ^20^ A reference set of conventional and melanotic schwannomas was included in the newly developed DNA methylation-based classifier tool for brain tumours.^22^ Additional studies are needed to clarify if the SH3PXD2A-HTRA1 fusion or any other molecular alteration in VS has prognostic relevance.

### Pathogenesis

Inactivation of the tumor suppressor gene NF2 plays a major role in the genesis of conventional schwannomas. A recent exome sequencing study demonstrated that 77% of VSs show evidence of genomic NF2 inactivation via loss of chromosome 22q or mutation of the NF2 gene.^23^ NF2 inactivation is the most common genomic alteration in VS. Biallelic inactivation can be demonstrated by exome sequencing in 45% of cases, whereas in 41% of cases only 1 allele affected by deletion of the heterozygous chromosome 22q or NF2 mutation is evident. In 14% of cases, no genomic impact on NF2 could be detected by exome sequencing. However, the consistent absence of merlin, the product of the NF2 gene, in VS tumor cells suggests that, in cases with no evidence of genetic inactivation, epigenetic mechanisms of NF2 silencing or mutational events in regions not covered by exome sequencing likely exist.^24^ Another recent whole-exome sequencing study reported concordant results regarding NF2 changes in VS.^25^ However, there are discrepancies between both studies regarding changes in non-NF2 genes. While one study found the ARID1A (14%), ARID1B (18%), DDR1 (11%), TSC1 (9%), TSC2 (7%), CAST (8%), ALPK2 (8%), LZTR1 (8%), and Table 3 (3%) genes to be recurrently altered in VS, the other study only found recurrent somatic mutations in the CDC27 (11%) and USP8 (7%) genes.^23^ More studies are needed to clarify the role of non-NF2 gene mutations in the pathogenesis of schwannomas.

RNA sequencing identified a recurrent SH3PXD2A-HTRA1 fusion on chromosome 10 in approximately 10% of VS cases. The fusion was associated with male sex predominance and partially occurred in combination with NF2 inactivation.^23^ Although the exact biochemical consequences of SH3PXD2A-HTRA1 fusion expression have yet to be elucidated, activation of the MEK-ERK signaling pathway appears to be involved. Inactivation of both alleles of PRKAR1A by deletion and/or mutation is considered an important event in the pathogenesis of melanotic schwannoma.^26^ In addition, melanotic schwannomas typically present monosomies of chromosomes-1, -2, -17, and -22q, as well as variable whole chromosome gains.^15^, ^20^

### Neurofibromatosis 2

Although VSs are typically solitary tumors, approximately 4%–6% of cases are associated with NF2. NF2 is an autosomal dominant monogenic condition caused by pathogenic variants on chromosome 22q of the NF2 gene.^27^, ^28^ Its incidence is of approximately 1:25,000 to 1:33,000, with a diagnostic prevalence of approximately 1 in 60‒70.^27^, ^29^ In the series by Evans et al.,^30^ only 7 out of 296 patients with NF2 had neither an affected parent nor other tumors suggestive of the condition. In rare cases, schwannomatosis caused by pathogenic variants in the Leucine-Zipper-Like Transcriptional Regulator 1 (LZTR1) gene may cause isolated VS that can be misdiagnosed as NF2. Optimal management includes screening of at-risk populations, early diagnosis, close surveillance, and development of treatment strategies based on the natural history of each associated feature (Table 1, Table 2 – adapted from Asthagiri et al.^31^).

**Table 1:** Patients at risk of neurofibromatosis type 2.

First-degree relative with neurofibromatosis type 2 (affected parent, sibling, or children)
People under 3 years of age with a unilateral vestibular schwannoma or meningiomas
People with multiple spinal tumors (schwannomas, meningiomas)
People with cutaneous schwannomas

**Table 2:** Recommended intervals for screening children of an affected parent.

Ophthalmological examination yearly from infancy
Neurological examination yearly from infancy
Audiology with auditory brainstem evoked potentials yearly from infancy
Presymptomatic genetic testing; one test from 10 years of age^a^
Cranial magnetic resonance imaging (MRI) at 10–12 years of age^a^
Spinal MRI at 10–12 years of age (every 2–3 years)

a Before 10 years of age in severely affected families and families for which early detection would aid in the preparation for future events related to neurofibromatosis type 2.

NF2 is diagnosed when the patient meets the criteria in Table 1 or when a pathogenic mutation in the NF2 gene is identified in the constitutional DNA or in two anatomically distinct tumors.^28^ Approximately 85% of NF2 cases initially present with bilateral VS,^32^ but unilateral VS with other features of NF2 may be present in up to 15% of cases.^28^, ^30^, ^33^ In addition, pathogenic LZTR1 variants may also present with an apparently solitary VS at young ages, particularly <25 years.^34^ The first members of a family to be affected, and particularly those with unilateral presentation, often (30%–35%) have mosaicism, as it occurs during early embryogenesis and was not present in the gamete.^34^

NF2-related VSs are typically multifocal and caused by different clonal events or multiple second hits that affect the NF2 gene in the Internal Auditory Canal (IAC), appearing along both branches of the vestibular nerve.^35^, ^36^ This makes surgery and other interventions such as radiation treatment more difficult, with higher recurrence rates.^37^ Radiation should be used with caution in young patients with NF2 due to the risk of malignant transformation and secondary tumor induction.^37^, ^38^ Although the course of NF2 is highly variable, strong genotype-phenotype correlations have been found between variants on exons 2–13 and reduced life expectancy.^39^ NF2 can cause schwannomas in the entire central and peripheral nervous systems. Patients may also develop spinal meningiomas and ependymomas. The associated morbidity severely affects Quality of Life (QoL) and reduces life expectancy (Table 3).^38^, ^39^

**Table 3:** Diagnostic criteria for Neurofibromatosis type 2 (NF2).

Definition of NF2			
**A**	Bilateral Vestibular Schwannomas (VS)		
**B**	Family history of NF2	PLUS	Unilateral VS or any 2 of: meningioma, glioma,* neurofibroma, schwannoma, posterior subcapsular lenticular opacities
**C**	Unilateral VS	PLUS	Any 2 of: meningioma, glioma,* neurofibroma, schwannoma, posterior subcapsular lenticular opacities
**D**	Multiple meningioma (2 or more)	PLUS	Unilateral VS or any 2 of: meningioma, glioma,* neurofibroma, schwannoma, posterior subcapsular lenticular opacities

NF2 should be considered in patients under 30 years of age with unilateral VS or other sporadic schwannomas and in patients under 25 years of age with a meningioma.^34^ Germline pathogenic mutations can be identified in 1%–10% of cases. NF2 should also be considered in older adults with two NF2-related tumors. Although germline detection rates are low, mosaic NF2 mutations can be confirmed if two identical pathogenic NF2 mutations are present in different tumors.^40^

## Objective

The objective of this study was to review the literature on the diagnosis and treatment of VS.

## Methods

On March 18, 2023, a task force consisting of otolaryngologists, otology specialists, Brazilian Society of Otology (*Sociedade Brasileira de Otologia*, SBO) directors, and SBO members met in person and remotely to discuss the topic of this guideline. Each participant in this meeting was tasked with giving a 15-minute evidence-based lecture on one of the suggested topics. After the lecture, the participants discussed the topic until reaching a consensus. Each author was asked to write a text with the current literature on the topic, based on evidence and containing the elements discussed during the meeting. A rapporteur prepared the final text, which was reviewed by four additional coauthors and the Brazilian Journal of Otorhinolaryngology (BJORL) editor.

This guideline is not intended to be a substitute for individual professional judgment. Physicians should always act and decide in a way that they believe is best for their patients, regardless of guideline recommendations. They should also operate within their scope of practice and in accordance with their training. The guidelines represent the best judgment of a team of experienced physicians addressing the scientific evidence for VS.

The grading system of the American College of Physicians (ACP) was used in this guideline, relating to critical appraisal and recommendations on therapeutic interventions^41^ (Table 4, Table 5). An important component of this guideline was judged to be critical appraisal of diagnostic testing studies. However, the ACP guideline grading system was not designed for this purpose.^42^, ^43^, ^44^

**Table 4:** Interpretation of the American College of Physicians’ Guideline Grading System (for therapeutic interventions).

Recommendation	Clarity of risk/benefit	Implications
**Strong recommendation**	Benefits clearly outweigh harms and burdens, or vice versa.	**Patients:** Most would want course of action; a person should request discussion if an intervention is not offered.
		**Clinicians:** Most patients should receive the recommended course of action.
		**Policymakers:** The recommendation can be adopted as policy in most circumstances.
**Weak recommendation**	Benefits closely balanced with harms and burdens.	**Patients:** Many would want course of action, but some may not; the decision may depend on individual circumstances.
		**Clinicians:** Different choices will be appropriate for different patients; the management decision should be consistent with patients’ preferences and circumstances.
		**Policymakers:** Policymaking will require careful consideration and stakeholder input.
**No recommendation**	Balance of benefits and risks cannot be determined.	Decisions based on evidence cannot be made.

**Table 5:** Recommendations (for therapeutic interventions) based on strength of evidence.

Recommendation and evidence of quality	Description of supporting evidence^a^	Interpretation
**Strong recommendation**		
High-quality evidence	RCT without important limitations or overwhelming evidence from observational studies	Can apply to most patients in most circumstances without reservation
Moderate-quality evidence	RCT with important limitations or strong evidence from observational studies	Can apply to most patients in most circumstances without reservation
Low-quality evidence	Observational studies/case studies	May change when higher-quality evidence becomes available
**Weak recommendation**		
High-quality evidence	RCT without important limitations or overwhelming evidence from observational studies	Best action may differ based on circumstances or patients’ values
Moderate-quality evidence	RCT with important limitations or strong evidence from observational studies	Best action may differ based on circumstances or patients’ values
Low-quality evidence	Observational studies/case studies	Other alternatives may be equally reasonable
**Insufficient**	Evidence is conflicting, of poor quality, or lacking	Insufficient evidence to recommend for or against

a This description of supporting evidence refers to therapy, therapeutic strategy, or prevention studies. The description of supporting evidence is different for diagnostic accuracy studies. RCT, Randomized Controlled Trial.

The American Thyroid Association (ATA) created a diagnostic test appraisal system that used the following methodological elements: consecutive recruitment of patients representative of clinical practice, use of an appropriate reference gold standard, directness of evidence (target population of interest, testing procedures representative of clinical practice, and relevant outcomes), precision of diagnostic accuracy measures (confidence intervals for estimates such as sensitivity and specificity), and consistency of results across studies using the same test that was also used in this guideline^43^ (Table 6, Table 7).

**Table 6:** Interpretation of the American Thyroid Association Guideline for diagnostic tests.

Recommendation	Accuracy of diagnostic information versus risks and burden of testing	Implications
**Strong recommendation**	Knowledge of the diagnostic test result clearly outweighs risks and burden of testing or vice versa.	**Patients:** In the case of an accurate test for which benefits outweigh risks/burden, most would want the diagnostic to be offered (with appropriate counseling). A patient should request discussion of the test if it is not offered. In contrast, for a test in which risks and burden outweigh the benefits, most patients should not expect the test to be offered.
		**Clinicians:** In the case of an accurate test for which benefits outweigh risks/burden, most patients should be offered the diagnostic test (and provided relevant counseling). Counseling about the test should include a discussion of the risks, benefits, and uncertainties related to testing (as applicable), as well as the implications of the test result. In contrast, for a test in which risks and burden outweigh the perceived benefits, most patients should not be offered the test, or if the test is discussed, the rationale against the test should, for the particular clinical situation, be explained.
		**Policymakers:** In the case of an accurate test for which benefits outweigh risks/burden, availability of the diagnostic test should be adopted in health policy. In contrast, for a test in which risks and burden outweigh the perceived benefits, some restrictions on circumstances for test use may need to be considered.
**Weak recommendation**	Knowledge of the diagnostic test result is closely balanced with risks and burden of testing	**Patients:** Most would want to be informed about the diagnostic test, but some would not want to seriously consider undergoing the test; a decision may depend on the individual circumstances (*e.g.*, risk of disease, comorbidities, or other), the practice environment, feasibility of optimal execution of the test, and consideration of other available options.
		**Clinicians:** Different choices will be appropriate for different patients, and counseling about the test (if being considered) should include a discussion of the risks, benefits, and uncertainties related to testing (as applicable), as well as the implications of the test result. The decision to perform the test should include consideration of the patients’ values, preferences, feasibility, and the specific circumstances. Counseling the patient on why the test may be helpful or not, in her/his specific circumstance, may be highly valuable in the decision-making process.
		**Policymakers:** Policymaking decisions on the availability of the test will require discussion and stakeholder involvement
**No recommendation**	Balance of knowledge of the diagnostic test result cannot be determined.	Decisions on the use of the test based on evidence from scientific studies cannot be made.

**Table 7:** Recommendations (for diagnostic interventions) based on strength of evidence.

Recommendation and evidence of quality	Methodologic quality of supporting evidence	Interpretation
**Strong recommendation**		
High-quality evidence	Evidence from one or more well-designed nonrandomized diagnostic accuracy studies (*i.e.*, observational — cross-sectional or cohort) or systematic reviews/meta-analyses of such observational studies (with no concern about internal validity or external generalizability of the results)	Implies the test can be offered to most patients in most applicable circumstances
Moderate-quality evidence	Evidence from nonrandomized diagnostic accuracy studies (cross-sectional or cohort), with one or more possible limitations causing minor concern about internal validity or external generalizability of the results	Implies the test can be offered to most patients in most applicable circumstances without reservation
Low-quality evidence	Evidence from nonrandomized diagnostic accuracy studies with one or more important limitations causing serious concern about internal validity or external generalizability of the results.	Implies the test can be offered to most patients in most applicable circumstances, but the utilization of the test may change when higher-quality evidence becomes available.
**Weak recommendation**		
High-quality evidence	Evidence from one or more well-designed nonrandomized diagnostic accuracy studies (*i.e.*, observational — cross-sectional or cohort) or systematic reviews/meta-analyses of such observational studies (with no concern about internal validity or external generalizability of the results)	The degree to which the diagnostic test is seriously considered may differ depending on circumstances or patients’ or societal values
Moderate-quality evidence	Evidence from nonrandomized diagnostic accuracy studies (cross-sectional or cohort), with one or more possible limitations causing minor concern about internal validity or external generalizability of the results	The degree to which the diagnostic test is seriously considered may differ depending on individual patients’/practice circumstances or patients’ or societal values
Low-quality evidence	Evidence from nonrandomized diagnostic accuracy studies with one or more important limitations causing serious concern about internal validity or external generalizability of the results	Alternative options may be equally reasonable.
**Insufficient**	Evidence may be of such poor quality, conflicting, lacking (*i.e.*, studies not done), or not externally generalizable to the target clinical population such that the estimate of the true effect of the test is uncertain and does not permit a reasonable conclusion to be made	Insufficient evidence exists to recommend for or against routinely offering the diagnostic test.

## Results/discussion

### Diagnosis

In addition to CN VIII, other CNs located within or close to the IAC may be compromised to different degrees (depending on VS growth) due to compression or reduced blood flow (Table 3). As tumor volume increases, symptoms such as headache (secondary to intracranial hypertension), cerebellar ataxia, hemiparesis, and involvement of the medullary nerves, leading to hydrocephalus (perceived by neurological and respiratory signs or by a papillary edema on eye fundus examination), evolve and may progress to a potentially fatal condition.^32^, ^45^

In general, the main VS-related complaints that guide the performance of additional tests are unilateral SNHL, tinnitus, increased deafness, vertigo, facial paralysis, hemifacial spasm, and hyposensitivity of the external acoustic meatus.^32^

### Audiometric tests

Pure-tone audiometry is the first additional test to be conducted. Several studies have created protocols for the diagnosis of VS based on clinical suspicion.^46^, ^47^, ^48^, ^49^, ^50^ Most of these protocols are notable for having good sensitivity but low specificity. In a meta-analysis conducted specifically to investigate the diagnostic accuracy (defined as the essential combination of sensitivity and specificity) of these screening protocols, the authors concluded that most studies were of poor-to-moderate quality.^51^ This may pose a problem for health systems because of the relatively low prevalence of VS in the population. MRI identified VS in only 4% of patients with sudden SNHL.^52^ Wilson et al.^53^ estimated that, in the US, the average cost of diagnosing a patient with VS based on MRI can reach US$ 61,650; considering the large number of patients who undergo the test and have normal results.

Bhargava et al.^46^ found that audiometric protocols had a sensitivity greater than 85% and a specificity ranging from 22% to 83% for diagnosing VS. Gheorghe et al.^48^ reported sensitivity ranging from 73% to 93%, but specificity ranging from 31% to 60%. Moffat et al.^47^ observed lowering of tonal thresholds at high frequencies in 56% of exams, cophosis in 25.5%, flat lowering of pure tone thresholds in 14%, and a “*U*” curve in 1.5%. Day et al.^49^ found a greater trend of correlation between tumor size and audiographic configuration, especially for tumors larger than 2.5 cm. Tests assessing the stapedial reflex, presence of recruitment, and speech discrimination may also assist in VS diagnosis.

The higher the sensitivity, the higher the number of detected VSs; the higher the specificity, the lower the number of missed VSs. The highest diagnostic accuracy was achieved by the American Academy of Otolaryngology-Head and Neck Surgery (AAO-HNS) protocol,^54^ which recommends MRI screening for patients with a mean asymmetry of ≥ 15 dB at frequencies of 0.5 kHz–3 kHz. This protocol has a sensitivity of 90.9% and a specificity of 57.5%. The protocol described by Gimsing^55^ recommends MRI for patients with a mean asymmetry of ≥15 B at frequencies of 1 kHz–8 kHz (except 3 kHz); it has slightly lower sensitivity and specificity than the AAO-HNS protocol (89.2% and 43.8%, respectively). None of these screening protocols was able to diagnose all patients with VS.

Other audiologic findings are worse predictors than audiograms. Absence of stapedial reflexes occurs more or less equally in patients with VS and in those without any tumors. Loss of speech discrimination occurs more frequently in patients with VS; however, several patients have a loss < 10% in this parameter without VS.

### Electrophysiologic tests

Vector electronystagmography can also be used for VS screening. Itani et al.^50^ found abnormal eye tracking test and optokinetic pattern results to be correlated with tumor size within the IAC. Day et al.^49^ reported that VSs larger than 2.5 cm are strongly correlated with abnormal caloric test results and altered cervical Vestibular Evoked Myogenic Potentials (VEMPs). Moffat et al.^47^ observed that caloric tests were normal in 86% of patients with VS.

Blödow et al.^56^ investigated changes in Vestibulo-Ocular Reflex (VOR) using caloric tests and Video Head Impulse Test (vHIT) in patients with VS (69 patients with a mean age of 58.1 years) and compared the methods in terms of sensitivity and specificity to detect retrocochlear lesions. They observed that unilateral hyporeflexia > 25% (in caloric tests), mean gain < 0.79 or gain asymmetry ratio > 8.5% (in vHIT), and fixation saccades were considered abnormal. The overall sensitivity of the caloric test was 72%, and the larger the tumor, the greater the hyporeflexia. When a cutoff of 50% was considered for unilateral hyporeflexia, the sensitivity of the vHIT was 45%, and the specificity was 90% (positive and negative predictive values of vHIT were 0.94 and 0.42, respectively).

Fujiwara et al.^57^ investigated the factors influencing semicircular canal function, as evaluated by vHIT, in patients with VS and found that the functions of all 3 semicircular canals on the side affected by VS were significantly lower than those on the unaffected side. Although there were no significant correlations between semicircular canal function and age, tumor size, and disease duration, a negative significant correlation between VOR gain, as evaluated by vHIT, and hearing loss was observed.

VSs can often present with normal Otoacoustic Emissions (OAEs) and cochlear feedback and abnormal Auditory Brainstem Response (ABR), mimicking a form of auditory neuropathy.^58^ ABR was used as a preliminary diagnostic method to select patients at high risk for VS prior to MRI examination.^58^, ^59^ The ABR using wave I–V latency differences was an important component of the clinical test battery for VS. Early studies claimed that the detection rates ranged from 95% to 98%, but the detected tumors were typically large. The combination of 2 ABR measures leading to interaural wave V delay and interpeak wave I to wave V delay detected medium and large tumors very well but often missed small tumors (<1 cm).^58^

Daniels et al.^60^ conducted a retrospective study in which patients with a tonal threshold > 70 dB were considered “ABR testable”. If ABR findings were normal, yearly audiometric follow-up could be requested for up to 5-years. However, if latency differences were found, further evaluation with contrast-enhanced MRI could be requested. It should be noted that the ABR test is less sensitive and specific than MRI for detecting tumors smaller than 1 cm.^58^ In the study by Urben et al.,^61^ 179 out of 325 participants underwent ABR testing and only 15 (8.3%) of them had abnormal results. MRI and ABR studies over the last 10–15 years have repeatedly confirmed these results and demonstrated that ABR tests miss 30%–50% of small tumors.^58^

Mangham^62^ showed that ABR alone (with a wave V latency difference > 0.2 ms) is more cost-effective than ABR in association with rotational vestibular tests. Koors et al.^63^ conducted a meta-analysis of 43 studies including 3314 patients undergoing ABR testing for VS diagnosis. ABR sensitivity was 93.4% (95% CI 92.6–94.3, *p* = 0.0000), and ABR sensitivity to detect extracanalicular tumors was higher than for intracanalicular tumors. Another recent review of nonimaging screening methods for VS found that, in 5 studies testing ABR, sensitivity and specificity values ranged from 37% to 100% and from 57% to 96%, respectively, which were not accurate enough for prescreening before MRI.^51^

#### Recommendations

I – Patients with asymmetric SNHL should undergo VS screening with MRI. Strong recommendation. Moderate degree of evidence.
II – In asymmetric SNHL, MRI should be performed regardless of ABR results. Strong recommendation. Moderate degree of evidence.
III – Vestibular tests may be conducted in patients with suspected VS, but normal results do not exclude the need for MRI. Weak recommendation. Low degree of evidence.
IV – Patients with asymmetric SNHL should undergo ABR testing alone, without the need for MRI. Not recommended. Insufficient evidence.

### Imaging tests

#### Evidence for suspecting and requesting tests for VS diagnosis

Gadolinium-enhanced T1-weighted MRI (GdT1w) is the gold standard for the detection and postoperative follow-up of recurrent and residual tumors.^51^, ^64^ The complete imaging study is performed using pre-contrast T1-weighted (T1w) and T2-weighted (T2w) sequences.^64^ The tumor is isointense on T1w and hyperintense on T2w.^65^ Intracanalicular VSs are cylindrical in shape, but extend from the porus acusticus into the cistern in a “drop” or “ice cream cone” configuration.

Gadolinium promotes an intense and homogeneous uptake by lesions. However, this uptake is more heterogeneous in larger lesions, allowing the identification of cystic lesions and necrosis.^65^ Lesion size and uptake pattern are often related to the histologic subtype: homogeneous tumors are typically smaller and made of Antoni A tissue, whereas heterogeneous and cystic tumors are typically larger and made of Antoni B tissue, in addition to showing more hemosiderin deposits.^66^

A more economical and faster alternative would be the use of high-resolution T2w MRI without contrast, which includes Fast-Spin Echo (FSE) and steady-state gradient-echo sequences such as Fast Imaging Steady-State Employing Acquisition (FIESTA) and Constructive Interference in Steady State (CISS).^67^ A meta-analysis compared the use of T2w vs GdT1w for detecting VSs and found T2w to be a highly accurate diagnostic and monitoring tool.^68^ As for cost-effectiveness, T2w was also shown to be more cost-effective than GdT1w for investigating cases of asymmetric SNHL.^69^ Crowson et al.^70^ found similar cost-effectiveness results, as well as shorter exam time and no contrast use with T2w. However, T2w has lower accuracy for detecting rarer CPA disorders, such as malignant neoplasms and inflammatory or infectious conditions, as well as intralabyrinthine lesions and IAC lesions smaller than 2 mm.^65^, ^71^

Another MRI finding when assessing VS is suppression of the Cerebrospinal Fluid (CSF) signal when using the Fluid-Attenuated Inversion Recovery (FLAIR) sequence. Patients with unilateral VS typically present a more intense intracochlear signal on the side of the tumor. The increase in signal intensity is related to the increase in the perilymph protein content secondary to the tumor. The clinical importance of this finding is not entirely clear, but it may be associated with hearing outcomes after tumor resection, as the signal was shown to normalize in patients experiencing successful hearing preservation but remain abnormal in those who lost hearing.^72^

Diffusion-weighted imaging is useful for differentiating VSs from arachnoid or epidermoid cysts. At least one T2w sequence is mandatory to exclude potential brainstem disorders mimicking VS symptoms such as multiple sclerosis or glioma.^45^ The axial heavily T2w sequence with submillimeter resolution is the most important sequence to evaluate the vestibulocochlear nerve and its branches and to describe the nerve as a linear hypointense structure surrounded by hyperintense CSF within adjacent cisterns.^73^

**Figure 1:**
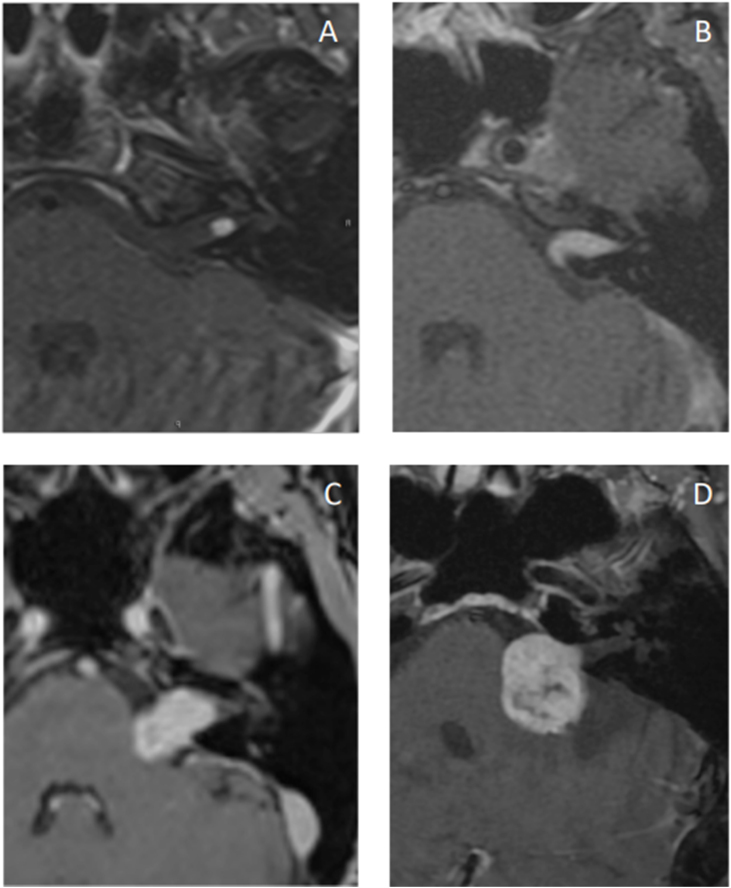
(A) Small intracanalicular tumor; (B) Small tumor with protrusion into the cerebellopontine angle; no contact with the brainstem; (C) Tumor occupying the cerebellopontine cistern with no brainstem displacement; (D) Large tumor with brainstem and cranial nerve displacement.

Although MRI is the gold standard for diagnosing VS, it has some limitations. First, patients with claustrophobia may find it difficult to undergo examination in conventional MRI machines (Fig. 1). Second, MRI ability to detect small tumors is lower in patients allergic to gadolinium or with poor renal function for whom contrast use is contraindicated. Finally, implantable metal prostheses, such as pacemakers and some types of hearing implants, may make the test unfeasible.^67^

VSs correspond to the majority (80%) of CPA tumors, followed by meningiomas (10%) and epidermoid cysts (<5%). Meningiomas typically appear as iso or hyperintense lesions on noncontrast-enhanced Computed Tomography (CT) and, in 20%–30% of cases, present calcifications, which is rare in VS. On MRI, they are isointense on both T1w and T2w. In some cases, the dura adjacent to the meningioma appears enhanced, and cases of “dural tail” enhancement have also been described, which can rarely be seen in VS.^74^ As for the shape, meningiomas are typically sessile and have a broad base against the petrous bone.

Epidermoid cysts may be indistinguishable from petrous bone cholesteatomas, especially those of congenital origin. Achieving an accurate diagnosis involves the use of FLAIR and diffusion imaging ‒ other imaging modalities may not be able to distinguish epidermoid cysts from VSs. Other schwannomas in the CPA are often associated with CNs V, VII, IX, X, XI, and XII and may be difficult to differentiate from VS radiologically in these cases, for better characterization, schwannoma location and the involved foramen should be identified. Other CPA lesions include metastases, vascular lesions (such as hemangiomas and other arteriovenous malformations), arachnoid cysts, inflammatory lesions, and other rarer ones.^65^

**Figure 2:**
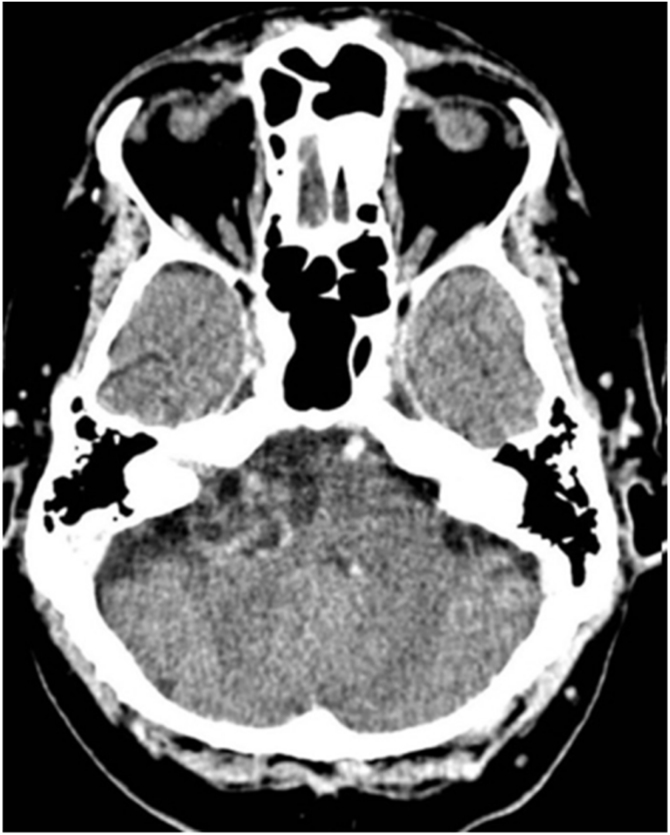
A large vestibular schwannoma in the right CPA.

CT was supplanted by MRI as MRI use became more common in the 1080s. However, it is still useful in patients for whom MRI is contraindicated or restricted, in addition to being cost-effective, fast, and well tolerated.^67^ VSs are typically isointense tumors that enhance with contrast.^75^ Although CT may identify bony remodeling of the IAC in larger VSs, it often misses lesions smaller than 2 cm.^67^ Other disadvantages include the risks of radiation and the use of iodinated contrast (Fig. 2).

#### Recommendations

V – Gadolinium-enhanced MRI is the gold standard exam for suspected VS. Strong recommendation. High degree of evidence.
VI – Patients already diagnosed with VS should undergo follow-up with T2w MRI without gadolinium-based contrast agents. Moderate recommendation. Moderate degree of evidence.
VII – For postoperative follow-up, patients should undergo gadolinium-enhanced MRI. Strong recommendation. Moderate degree of evidence.
VIII – The role of CT in the diagnosis of VS is limited. Not recommended. Insufficient evidence.
IX – CT can be used to assist in surgical planning. Weak recommendation. Low degree of evidence.

### Treatment

Treatment strategies vary greatly between centers and countries, and decision making has been complex. Tumor characteristics such as initial size and growth on serial imaging (often > 2 mm between scans) are commonly associated with the decision to start treatment. Surgery is the mainstay of treatment because it removes the tumor, allowing histologic evaluation. Radiotherapy is indicated only in selected cases. Adequate patient and family counseling is required.

#### Wait and scan protocols

The detection of a completely asymptomatic VS on imaging tests performed for other reasons, such as chronic headache, is a clinical challenge. Some studies have shown that small and asymptomatic VS tend not to grow, unlike larger and symptomatic ones.^76^ However, the best surgical results in terms of facial function and hearing preservation are obtained with small, barely symptomatic tumors.^77^, ^78^ Although the incidence of VS has not increased significantly over the past decade, small VSs have been increasingly detected because of recent improvements in gadolinium-enhanced MRI techniques.^67^ This led to a change in the trend of VS management towards an increasingly conservative approach.

Tumor growth rate and duration of treatment do not seem to differ between small and asymptomatic VSs with a larger and symptomatic one. Only a few prognostic factors can predict tumor growth and symptom progression.^76^ The probability of hearing loss over time, even with a small decline (1%–10%) in the speech recognition index, is significant. More than 50% of patients lose functional hearing during the observation period.^79^ Hearing loss may continue to progress despite a lack of tumor growth.^80^ A recent retrospective analysis of the US database SEER revealed that the number of VS managed with the “wait and scan” approach increased over time, especially in older patients with smaller tumors. It also predicted that, by 2026, half of all VS cases will be managed initially with observation alone.^81^

One of the main arguments supporting observation is that in approximately 58%–71% of small VSs, tumor size is stable over time.^82^ Nikolopoulos et al.^83^ conducted a systematic review of VS growth and found that up to 75% of tumors (ranging from 6% to 75%) did not grow during the study period (ranging from 19 months to 5.5 years). Tumor growth does not necessarily require an intervention. The failure of conservative treatment for intracanalicular VSs in studies with a 10-year follow-up is approximately 15%.^84^ A retrospective study reported that a tumor size greater than 7 mm at diagnosis was associated with an increased risk of tumor growth during observation.^82^ There is no agreement of larger tumor size at diagnosis and higher risk of growth.^51^, ^85^, ^86^

The symptoms is not necessarily a predictor of initial tumor size, although incidentally discovered VS tend to be smaller than symptomatic ones.^2^, ^86^ Known predictors of VS growth include IAC filling, cystic and hemorrhagic features within the tumor, hormone treatment, extracanalicular component greater than 20 mm, young age at diagnosis, and NF2. Some authors suggest adopting a “watch and wait” approach for small, asymptomatic lesions and switching to active treatment in case of tumor growth greater than 2–3 mm per year and/or significant worsening of symptoms.^87^, ^88^, ^89^ Small VS have better postoperative functional results compared with larger ones. Smaller tumor size is a well-known positive prognostic factor for preservation of both facial nerve and hearing function (Table 8).^77^, ^89^, ^90^

**Table 8:** Neurological signs and symptoms according to Cranial Nerve (CN) involvement after vestibular schwannoma growth.

Cranial nerve	Symptoms	Signs on physical examination
Trigeminal nerve (CN V)	Pain on one side of the face	Loss of corneal reflex
Facial nerve (CN VII)	Facial paralysis	Different degrees of facial paralysis
Intermediate nerve of Wrisberg	Taste alteration, dry eyes, and decreased tearing	Abnormal Schirmer test, reduced sensitivity in the Ramsay Hunt’s zone (Hitzelberger’s sign)
Glossopharyngeal nerve (CN IX) and vagus nerve (CN X)	Hoarseness and gagging	Vocal alterations, asymmetric soft palate, aspiration, or penetration on swallow test

Conservative management is not a viable option for large VSs, nor is radiotherapy, especially in the presence of a mass effect.^91^, ^92^ Studies on the use of radiotherapy in patients with large VSs that are not candidates for surgery^93^ reported different results, with the rates of tumor control being directly associated with tumor size.

There are several grading systems for tumor size that support decision making,^91^, ^94^ of which the Koos classification is the most commonly used (Table 9).^94^

**Table 9:** The Koos grading system.^94^

Koos grade	Tumor description
I	Small intracanalicular tumor
II	Small tumor with protrusion into the cerebellopontine angle; no contact with the brainstem
III	Tumor occupying the cerebellopontine cistern with no brainstem displacement
IV	Large tumor with brainstem and cranial nerve displacement

When comparing observation, radiotherapy, and microsurgery for small VSs in relation to auditory function, in the short term, hearing preservation is better in patients undergoing conservative management than in those undergoing active treatment (either surgery or Stereotactic Radiotherapy [SRT]).^89^, ^95^ However, in the long term, some studies showed that, after 2 years of follow-up, the decline in hearing function was faster in patients undergoing observation, while predictably remaining stable over time after surgery, as assessed at 10 and 15 years.^96^ Despite studies indicating that these tumors grow within the first 5 years of follow-up, imaging follow-up should continue beyond this period because they may continue to grow slowly and could be unpredictable over time.^2^ Patient adherence to recommendations should be taken into account, as nonadherence may lead to failure to follow-up.^97^ The goal of observational management is to monitor tumor growth and hearing function to assist in the decision making regarding choice of treatment.

#### Recommendation

X – Patients with asymptomatic, stable-growing tumors (< 2 mm/year) that remain asymptomatic over time may undergo observation alone, especially if Koos grade I. Therapeutic intervention may be offered to patients with stable tumors as well as progressive SNHL and/or vestibular symptoms that are disabling or refractory to clinical treatment. Strong recommendation. Low degree of evidence.
XI – Intervention is indicated for tumors with significant growth (≥2 mm/year) and/or on Koos grade III and IV. Strong recommendation. Low degree of evidence.
XII – “Wait and scan” patients with NF2-related VS should undergo MRI every 6 months, especially those with bilateral VS, because of the increased risk of tumor growth compared with sporadic VS. Strong recommendation. Low degree of evidence.

### Surgery

The decision to surgically treat a VS should be based on individual patient characteristics, such as age, previous operations, and size/position of the tumor. Audiologic assessment and experienced symptoms can help decide on the best surgical approach for a given patient.^98^ For large VSs (Koos grade IV), surgery is considered the primary treatment for removal of tumors with a potentially fatal mass effect.^94^ Surgery can also be considered for smaller tumors in case of cystic degeneration or if cure is the main goal of treatment.^99^, ^100^

#### Intraoperative monitoring

The goal of surgical VS management, in addition to removing the tumor, is to cause the least possible injury to offer the patient a better QoL. Therefore, monitoring of the CNs, especially the facial nerve, is commonly performed; other CNs can also be monitored according to the needs and preferences of each surgical team.

#### Anesthetic procedure

Several anesthetic agents can affect intraoperative neurophysiological monitoring. Inhalational anesthetics have the greatest effect and should be avoided, as they decrease the amplitude and increase the latency of evoked responses. Intravenous anesthetics, such as propofol and opioids, are considered the agents of choice. Care should be taken when performing infiltration in the mastoid tip region, as it may hinder or even make monitoring of the facial nerve unfeasible for several hours.

Muscle relaxants should also be avoided because they inhibit muscle contractions, but if their use is required, short-acting drugs such as succinylcholine can be administered, as their effects disappear before monitoring begins. Current anesthetic techniques allow intubation without the use of neuromuscular blockers and with adequate depth of anesthesia.

Some physiological parameters controlled by the anesthesiologist during the procedure may affect neurophysiological monitoring, often by increasing latency or decreasing amplitude. Hypotension (reduced cerebral blood flow) and hyperventilation (causing hypocapnia) can lead to cerebral vasoconstriction, whereas excessive bleeding and anemia can cause hypoxemia and hypothermia. All of these events can slow nerve conduction velocities.^101^

#### Facial nerve

Facial paralysis has potentially devastating functional effects, as well as emotional and social consequences for the patient. The last 30 years have seen a progressive improvement in facial nerve preservation after surgical resection of CPA tumors.^102^ Several methods for intraoperative monitoring of the facial nerve have been developed. Electromyography (EMG) is considered the gold standard.

#### EMG

There are several important parameters that complement each other, such as transcranial Motor Evoked Potentials (MEPs), continuous free-running EMG, and stimulated EMG. However, only continuous EMG provides real-time information. In addition, the neurophysiologist does not need to interrupt the procedure to perform motor stimulation, nor does the surgeon to perform probe stimulation.

Stimulated EMG and MEPs rely on measurements of the Compound Muscle Action Potential (CMAP) generated by the muscles of facial expression. On continuous EMG, the observed discharges are motor unit potentials that depolarize asynchronously due to membrane irritation by mechanical traction or thermal or chemical irritation. They are desynchronized and spontaneous. CMAP involves depolarizing a large pool of motor units in a synchronous fashion. This is what happens when there is stimulation by the cerebral cortex or the probe. Depolarization of the facial nerve leads to distal propagation of nerve action potential to the motor endplate, where it is translated into motor unit potentials emanating from the corresponding muscle fibers.^103^

An accurate assessment of nerve conduction with EMG requires stimulation proximal to the potential site of injury. When an electrical stimulus is applied distally to the injury during the surgery, a seemingly normal response may be obtained. Wallerian degeneration of distal axons following severe nerve injury takes 48–72 h to reach the motor endplate. MEPs are important because they assess the entire motor pathway and detect injuries intraoperatively. Nerves suffering from mild-to-moderate injury will exhibit reductions in amplitude and prolonged latency both on MEPs and stimulated EMG. Increasing injury requires an increasing amount of current to elicit a response. A combination of physiological conduction block (neurapraxia) and physically injured neural elements (axonotmesis or neurotmesis) will often be evident after significant surgical trauma. These injuries will be variably represented on stimulated EMG and MEPs by reduction in amplitude, increase in latency, and increase in threshold stimulation as the level of nerve injury increases.^103^, ^104^ Combined audio and visual feedback allows the monitoring team to be as vigilant as possible in regard to changes in nerve status, both by the surgeon and the monitoring physician.

#### Facial motor evoked potential

Facial MEPs (FMEPs) provide an intermittent functional assessment (stimulus triggered from time to time) of the facial nerve during surgery. If FMEPs decline more than 50%–60% compared with the baseline during tumor dissection and do not recover after a pause, further dissection should be avoided. FMEPs allow monitoring of the facial nerve before it is identified, especially in the early stages and in large tumors.^105^ Additional surgical procedures can be performed (such as pausing, irrigating the nerve, changing the dissection area) before significant irreversible injury occurs,^106^ and peripheral MEPs should be used to monitor the patient.^103^ An FMEP ratio of 0.60 is a predictor of satisfactory facial nerve function at 1-year and indicates functional/physiological integrity of the nerve during surgery.

#### Electrocautery precautions

EMG monitoring is disabled during the use of electrocautery, as electrocautery generates a high-intensity electrical artifact that typically overpowers the ability of the monitor to record low amplitude EMG activity. Thermal nerve injury due to electrocautery may not be detected until after the injury has occurred and then only if the nerve has been stimulated to assess its function. Baseline stimulation should be performed as early and as often as possible using moderate-level mapping currents prior to initiating tumor dissection. Stimulation is particularly important before and after any risky surgical procedure to ensure appropriate function of the monitoring system and to detect nerve injury at the earliest time possible.^103^

#### Adequate electrode placement

For facial nerve monitoring, intramuscular needle electrodes are inserted in a closely paired manner at the nasolabial groove and near the eyebrow on the side to be monitored (basic placement, other branches may be used). The impedance of each independent electrode should be less than 5 kOhm, whereas interelectrode impedance should be less than 2 kOhm. If impedance is too high, it might be because of poor needle position or faulty electrodes. The electrodes should be repositioned or replaced and then retested.^103^

#### Current intensity

A normal facial nerve will respond to a stimulation of at least 0.05 mA when the probe is placed directly on the nerve in the CPA. However, with increasing distance, as well as intervening soft tissue, bone, CSF, or blood, a current of 1–2 mA may be required to obtain a baseline “far-field” response by volume conduction of current through tissue.^103^

#### Prognosis

Postoperative facial function results reported in published studies are long-term, evaluated at least 6-months after surgery. Immediate facial function will inevitably be worse due to the acute effects of tumor dissection. Neurapraxia will resolve in a matter of weeks, but axonotmesis is more variable because it depends on motor neuron reinnervation of denervated muscle fibers. Although the reinnervation process can take 6–18 months, it is likely that there will be some improvement in facial function. Patient anxiety is compounded by the lack of information. It is important to predict the speed and degree of recovery.

Patients with immediate postoperative facial paralysis will experience gradual improvement, assuming the anatomical integrity of the facial nerve was preserved. One-year follow-up is advisable in patients with unsatisfactory facial function to allow adequate time for full recovery to occur.^105^ The intraoperative minimal stimulation threshold is a valuable prognostic indicator of long-term facial function. It assesses the minimum current required to evoke a muscle response after tumor resection.

#### Cochlear nerve

ABRs are widely used for intraoperative monitoring of cochlear nerve and brainstem function. The different ABR patterns are identified and correlated with the postoperative audiologic results of VS surgery.^107^ Interpeak intervals are more informative than latencies, as latency is more influenced by age and other external factors. Loss of wave V combined with prolonged latency (≥1 ms) and a >50% reduction in wave V amplitude is a strong predictor of postoperative hearing loss. Decreased wave V amplitude is the best predictor of an abnormal ABR. The surgeon should be informed when a 46% decrease in amplitude occurs, and a 55% reduction suggests possible postoperative hearing loss.^108^

Another monitoring modality available is Cochlear Nerve Action Potential (CNAP), in which the electrode is placed directly on the proximal cochlear nerve in the CPA or IAC. This concept was first described by Silverstein et al. in 1985^109^ and remains virtually unchanged. The CNAP stimulus is the same as an acoustic ABR. The recorded neural amplitudes are measured in microvolts (μV) and are comparatively much larger than the ABR signal. The larger CNAP signal is secondary to an improved signal-to-noise ratio, as the recording electrode makes direct contact with the neural generator. The larger amplitude and clearer signal mean less averaging (10–300 trials) and, essentially, a real-time measurement. The CNAP response may be seen even in the absence of conventional ABR waveforms.^110^ The CNAP waveforms have 4 major vertexes: two with a negative deflection (N1, N2), and two with a positive deflection (P1, P2). The N1 negative vertex serves as the primary monitoring wave of interest. Although the CNAP signal is reliable, placement of the electrode can be challenging given the dynamic pulsations of the brain, CSF, and tumor dissection.^111^ Consensus on which CNAP waveform changes indicate a clinically meaningful difference is lacking. Absolute changes in N1 latencies are commonly used.^100^ Some studies indicate that CNAP is superior to ABR and leads to improved hearing preservation outcomes,^112^, ^113^ but some studies show no difference between ABR and CNAP.^114^ ABR and CNAP can be used simultaneously. Regardless of which technique is used, the surgeon and monitoring team should be aware of the advantages and disadvantaged inherent to each system.

VSs have minimal influence on OAEs if the hearing is normal.^115^ Preoperative OAEs have poor predictive value for hearing preservation surgery compared with other measured characteristics.^116^ Intraoperatively, OAEs present a rapid response to tumor and cochlear nerve manipulation, with some postoperative predictive value if they are lost.^117^

Electrocochleography (ECochG) is another marker of cochlear function and more sensitive than pure-tone audiometry. The measurement is a complex interaction of outer hair cell function measured by the cochlear microphonic, inner hair cell function measured by the summating potential, and proximal cochlear nerve function measured by the action potential.^118^ ECochG has been used to predict Cochlear Implant (CI) outcomes and hearing preservation.^119^, ^120^ The use of ECochG in VS and lateral skull base microsurgery has been limited thus far. A study by Riggs et al.^121^ using ECochG in Translabyrinthine (TLB) VS microsurgery showed variability in response measurements ranging from 0.1 Mv to 100 μV. The authors also identified different sites of hearing loss, with some cases showing a reduced summation potential suggestive of an intracochlear deficit, whereas other cases showed more neural detriments. Despite a moderate correlation (*r* = 0.67) of ECochG with preoperative Word Recognition Score (WRS), two patients with 0% WRS showed good cochlear function on ECochG, suggesting their SNHL had a neural etiology. The study suggests that predictive modeling with ECochG might provide information on lesion site and whether the cochlear nerve is healthy enough to carry the CI signal.

#### Electrically evoked ABR

The electrically Evoked Auditory Brainstem Response (eEABR) test is similar to the ABR, but it delivers electrical stimulus directly to the cochlea, which makes it more effective than ABR for quantifying nerve conduction of the auditory pathways.^122^ The eEABR can objectively measure CI function and peripheral auditory neurons/nerve responsiveness up to the level of the brainstem. In addition, these signals can be recorded even when excessive stimulus artifacts preclude successful acquisition of the electrically evoked compound action potentials.^123^ Like acoustic ABR, eEABR requires thousands of cycles to compute a reliable waveform. The electrical artifact also degrades waves I and III, making wave V the only reliable waveform in eEABR.^111^

CIs have their own telemetry function that can record spiral ganglion neural activity. In a case of unilateral, sporadic VS resection, neural response imaging was added to eEABR to provide real-time, near-field measurements.^124^ Another measurement of cochlear nerve integrity is the stapedial reflex. The feasibility of electrical stimulation of the stapedial reflex has been described as a complement to eEABR and telemetry.^125^ Further studies are needed to assess the full benefit of these techniques.

The MED-EL company (Innsbruck, Austria) has developed a stimulating system with a disposable electrode array. The Auditory Nerve Test System (ANTS) comprises three parts: auditory nerve test electrode, connector cable, and stimulator box.^126^ The ANTS has not been associated with perioperative complications. On-going studies are needed to determine the predictive value of intraoperative eEABR and the impact of deafness rehabilitation on QoL. CI outcomes following VS microsurgery should also account for baseline hearing characteristics measured by audiograms or ECochG.

Studies with better baseline hearing function have shown superior CI outcomes.^127^ The first published series using ANTS in TLB VS microsurgery with simultaneous CI reported on 5 patients with severe-to-profound SNHL and poor WRS.^128^ Another series of 14 patients conducted a similar assessment.^129^ The authors found that the eEABR data, when available, correlated with CI performance. Among patients with an eEABR, all received auditory perception with their CI. Only 1 of the 5 patients without an eEABR heard sound without a CI.

#### Vagus nerve monitoring

When the VS is large and extends significantly toward the jugular foramen, monitoring the vagus nerve via its Recurrent Laryngeal Nerve (RLN) should be considered. Similar to facial nerve monitoring, RLN monitoring is also based on EMG (free running, stimulated, and MEP). Currently, the most commonly used method of intraoperative RLN monitoring uses surface electrodes along an endotracheal tube. Vagal stimulation appears to be safe at levels of approximately 0.5–1 mA. It is recommended to begin with 0.5 mA and increase amplitude slowly, as needed.^103^

#### Recommendations

XIII – Facial nerve monitoring should be conducted during VS resection surgery. Strong recommendation. Moderate degree of evidence.
XIV – Cochlear nerve monitoring may be performed in patients who still have serviceable hearing. Strong recommendation. Moderate degree of evidence.
XV – Monitoring of other CNs (in addition to the facial nerve) can also be performed. Moderate recommendation. Low degree of evidence.

### Surgical approach

The choice of surgical approach should be individualized, and the surgeon should be able to perform different approaches based on indication criteria and personal experience. Treatment should be performed in high-volume centers. Surgery-related mortality is 0.5% in large series.^130^ The probability of hearing preservation in patients with normal hearing (Gardner-Robertson class A; Table 10)^131^ is >50%–75% immediately after surgery, as well as after 2 and 5 years, and >25%–50% after 10 years.^132^ Factors influencing the preservation of serviceable hearing after microsurgery are tumor size <1 cm, presence of a distal IAC CSF fundal cap, and good preoperative auditory function.^132^

**Table 10:** Gardner-Robertson scale for hearing function.

Grades	Pure tone audiogram (dB)	Speech discrimination (%)
I	0–30	70–100
II	31–50	50–69
III	51–90	5–49
IV	91–max	1–4
V	Not testable	0

The risk of persistent facial paralysis ranges from 3% to 46%;^133^, ^134^ it depends on tumor size and the occurrence of immediate paresis.^133^ Intraoperative monitoring in VS surgery is required and should include somatosensory evoked potentials and facial nerve monitoring with direct electrical stimulation and free-running EMG. Intraoperative monitoring of the facial nerve leads to a better functional outcome and can be used to accurately predict favorable facial nerve function after surgery.^132^ EABRs should also be used when trying to preserve hearing.^81^, ^135^ For large VSs, EMG of the lower CNs is recommended.

The goal of surgery should be total tumor resection, as residual tumor volume is correlated with the recurrence rate. In a series of 116 patients with VS who underwent Gross Total Resection (GTR), Near-Total Resection (NTR), or Subtotal Resection (STR), recurrence rates were 3.8%, 9.4% and 27.6%, respectively.^136^ The median time to recurrence was 22 months, ranging from 6 to 143 months. Jacob et al.^137^ reviewed 103 patients with sporadic VS who underwent NTR or STR and found that those who underwent STR were over 13 times more likely to recur compared with those who underwent NTR. In a study by Chen et al.,^138^ of 111 patients with incomplete excisions (NTR or STR), all 7 patients who showed evidence of residual tumor had undergone STR. Several other series have also shown a considerably higher risk of reoperation with greater residual tumor volumes.^139^, ^140^

In these cases, partial resection followed by Stereotactic Radiosurgery (SRS) (see below) has become increasingly popular.^141^, ^142^, ^143^ Published results on this combined approach show superior outcomes with regard to facial nerve function and hearing preservation when compared with total resection, with comparable tumor control rates. However, these studies are small and retrospective (class of evidence IV; good practice point).^141^, ^143^ After NTR or intentional STR, a “wait and scan” approach is warranted as only a minority of remnants progress; however, the risk increases with the size of the remnant.^143^ In cases of recurrence after radiosurgery, both reoperation and radiosurgical retreatment are possible. However, patients with previously irradiated tumors are at greater risk of poor facial nerve function after surgery, and a very meticulous and conservative dissection technique may be required (class of evidence: IV; good practice point). In patients with recurrent VS after surgery, radiosurgery is preferred because the risk of facial nerve injury is lower than with reoperation (class of evidence III; level of recommendation C).^144^, ^145^, ^146^

#### Suboccipital retrosigmoid (retromastoid)

The Retrosigmoid (RS) approach is a modification of the suboccipital approach. While the suboccipital access is located near the midline, the RS access is projected more anteriorly and laterally. It provides wide visualization of structures from the tentorium cerebelli to the foramen magnum. The limit of exposure is defined anteriorly by the sigmoid sinus and superiorly by the inferior border of the transverse sinus. It allows removal of tumors of various sizes and has the possibility of hearing preservation. It provides excellent visualization of the brainstem, CNs, and relevant vascular structures, but requires some cerebellar retraction and allows only limited access to the fundus of the IAC.^147^ The main advantages of the RS approach are the possibility of hearing preservation and adequate exposure of the lower structures of the CPA.^148^

The RS approach is indicated for VS resection when there is a possibility of hearing preservation and if the tumor extends less than 1 cm into the IAC. The indication depends on the level of preoperative hearing. Eligible patients must have a pure tone average > 50 dB and/or a speech discrimination score >50%. It can be performed for tumors of any size, especially extracanalicular ones.^149^

### Surgical technique

#### Patient positioning

The RS approach can be performed with the patient in supine, lateral supine (“park bench”), or semisitting position. The headrest (Mayfield®) is attached to the structure of the operating table. This position allows exposure of the suboccipital area when rotating the operating table. Although there are some small retrospective studies reporting the superior functional outcome associated with the semisitting position, current data do not support favoring any specific position.^150^, ^151^, ^152^ Excessive rotation of the neck should be avoided so as not to compromise the perfusion of the venous system. To safely rotate the operating table, a strap should be used to secure the patient to the table, and lumber support should be provided.

#### Incision and exposure of the dura mater

A curvilinear incision is made 3 cm from the retroauricular sulcus. The incision begins 1 cm above the pinna and extends inferiorly to 1 cm below the tip of the mastoid. Cervical muscles should be dissected from the mastoid and suboccipital region. Bleeding from the emissary veins is controlled with bone wax. Exposure should extend to the tip of the mastoid, and the retractors are then positioned. The incision is extended to the periosteum overlying the mastoid. An inverted L-shaped incision (or U-shaped incision based inferiorly) is made in the periosteum along the posterior edge of the mastoid and along the superior insertion of the occipital muscles.

The suboccipital muscles are elevated in the subperiosteal plane and are retracted medially and inferiorly. This expands the exposure inferiorly. The use of electrocautery can facilitate the dissection of the muscles adhered to the tip of the mastoid. Emissary vein bleeding should be controlled with bone wax before craniotomy.

#### Craniotomy and tumor exposure

An approximately 3 × 3 cm bony window is made in the posterior fossa. The sigmoid sinus corresponds to the anterior limit of the window, whereas the transverse sinus corresponds to the superior limit. Identification of the sigmoid sinus is facilitated by entry into mastoid air cells. Cutting and diamond burs can be used for craniotomy. Drilling can proceed along the sigmoid sinus toward the junction of the transverse and sigmoid sinuses. The most used technique is the drilling of 2 or 3 contiguous holes, which will be joined later. All opened air cells require obliteration with bone wax to prevent CSF leakage.

The bone flap is separated from the dura, removed, and stored in saline. The edges of the craniotomy should be enlarged and trimmed with cutting burs. Before creating the dural flap, mannitol and hyperventilation may facilitate brain relaxation. A small incision can be made in the dura mater to later complete the flap incisions. Several flaps have been described in the literature. A cottonoid can be placed between the dura and the cerebellum for protection while making the incisions. Later, the dural flaps can be anchored with sutures.

The patient is placed in the reverse-Trendelenburg position for CSF drainage and improved exposure. The inferior surface of the cerebellum is gently retracted superiorly over a cottonoid. The arachnoid of the cisterna magna is entered just inferior to the lower CNs, allowing CSF drainage. If difficulty relaxing persists, therapeutic lumbar puncture can be performed. These measures allow posterior fossa decompression, relaxing the cerebellum, which retracts medially.

At this point, the retractor should be positioned more anteriorly with slight retraction, thus allowing the arachnoid overlying the CPA to be separated from the lower CNs. This dissection should be performed from the tentorium to below the lower CNs. The dissection of the upper pole of the cerebellar hemisphere in relation to the tentorium allows full access to the uppermost portion of the CPA. Venous vessels should be carefully coagulated and divided. At this point, the trigeminal nerve can be identified superiorly to the tumor. The cerebellar flocculus may overlap the entry zones of CN VII and CN VIII. Therefore, they should be moved from the cerebellar peduncle and lateral surface of the pons. The Anterior Inferior Cerebellar Artery (AICA) and its branches should be identified inferiorly to CN VIII, and the Posterior Inferior Cerebellar Artery (PICA) close to the lower CNs. This exposure provides the identification of the tumor surface in the CPA and the CN VIII in the medial pole of the tumor.

#### IAC opening and tumor removal

For adequate exposure of the posterior wall of the canal, the operating table should be rotated, and the microscope repositioned. It is important to properly locate the IAC by palpation with a right-angled hook. It is recommended to place gelatin sponges (Gelfoam) at the lower and upper poles of the CPA. This measure prevents the spread of bone debris into the subarachnoid space.

The dura is coagulated with a bipolar electrocautery before incision. Several dural flaps have been described in the literature, but the most commonly used is the U-shaped flap. The incision with an 11-blade scalpel begins in the dura over the porus area, extending the incisions laterally. Then, a small Lempert elevator is used to detach the dura. Care should be taken when making the lower incision, as the jugular bulb may be dehiscent. Likewise, if the incision is too lateral, it may cause laceration of the sigmoid sinus. This flap is rotated posteriorly toward the tumor, providing protection during drilling of the IAC.

The IAC should be drilled from a medial to lateral direction in the line corresponding to the canal. The canal should be opened laterally as needed and according to the lateral extension of the tumor. A cutting bur can be used to start opening the IAC and removing excess bone overlying the canal. The thin residual layer is then dissected from the dura of the IAC before drilling with a diamond bur. In general, the exposure is limited to the medial two-thirds of the IAC. Exposing the lateral one-third of the canal may result in opening the common crus or the vestibule, thereby compromising hearing. It is estimated that exposure of up to 5 mm of the IAC is safe concerning inner ear function. In selected cases, it can be expanded up to 10 mm laterally. The extent of the tumor in the IAC can be previously assessed by gadolinium-enhanced MRI to define the need for exposure. After exposing the dura of the IAC, bony troughs should be created superiorly and inferiorly to facilitate tumor manipulation in relation to the IAC nerves and positive identification of the facial nerve. These troughs should be wider in the porus acusticus to provide a 180- to 270-degree exposure of the IAC circumference.

At this point, the dura overlying the IAC is incised along the canal with micro-scissors. To create dural flaps, a transverse incision should be made next to the porus acusticus, and another incision at the fundus of the IAC. These flaps are reflected superiorly and inferiorly and provide exposure of the tumor and IAC contents. In most cases, tumor dissection should begin at the IAC. Unlike the region medial to the porus acusticus, the facial nerve has a consistent position within the IAC. Therefore, positive identification is possible with electrophysiological monitoring of the facial nerve at the very beginning of tumor dissection.

Tumor dissection should begin laterally by identifying the plane between the facial nerve and the tumor. The use of a delicate dissector allows the tumor to be displaced inferiorly in the topography of the facial nerve. This maneuver provides wide lateral exposure of the facial nerve. After establishing this plane between the facial nerve and the tumor, a right-angled instrument can be used to dissect the tumor from the posterior surface of the cochlear and facial nerves. This maneuver allows the transection of the superior and inferior vestibular nerves with micro-scissors. The dissection of this plane should proceed toward the porus acusticus medially. However, care must be taken to avoid traumatic avulsion of the cochlear nerve and the fibers passing into the modiolus. It is important to monitor ABR changes during this maneuver. Even with anatomical preservation of the cochlear nerve, hearing preservation may not be possible. Interruption of blood supply to the cochlear nerve may occur at the CPA (by branches of the labyrinthine artery through an AICA loop) or at the IAC (by branches of the labyrinthine artery between the inferior vestibular nerve and the cochlear nerve).

The tumor capsule in the extracanalicular portion should be stimulated to check for an unidentified facial nerve under the capsule. A rectangular-shaped incision is made in the capsule with an 11-blade scalpel in the medial position to avoid that the bleeding flow from the uppermost portion compromises the surgical field. Material should be removed with micro-scissors for anatomopathological examination. The next step is internal debulking of the tumor. Debulking can be performed with ear forceps, cold dissection using micro-scissors, or ultrasonic aspirator. After volume reduction, the capsule can be more easily separated from the cerebellum, vessels, and nerves with the use of micro-scissors.

The medial tumor dissection plane begins posteriorly along the middle cerebellar peduncle. An arachnoid plane is defined and gradually developed on the lateral surface of the pons. At this point, it is possible to identify the CN VIII in the brainstem. The CN VIII enters the brainstem laterally just above the facial nerve. There is often a branch of the AICA between the facial nerve and CN VIII near the brainstem. If there is a chance of hearing preservation, the vestibular nerves should be separated from the cochlear nerve in the area proximal to the brainstem to provide a tumor dissection plane. Then, the facial nerve can be identified in the brainstem in a more anterior position on the surface of the brainstem. In general, the course of the facial nerve is anterior to the tumor and may be more superior or inferior. Tumor dissection should proceed from a medial to lateral direction, especially in small tumors.

Larger tumors do not allow for unidirectional dissection. Tumor rotation is often required to properly expose the facial nerve. The plane between the tumor and the nerves is followed to the porus acusticus and into the IAC. Small tumor fragments are often adhered to the facial nerve, preventing GTR. Therefore, NTR is possible and does not usually lead to tumor recurrence. Conversely, tumor remnants at the fundus of the IAC or near the brainstem can be a source of recurrence, as they receive vascular supply.

At the end of the procedure, it is important to perform ABR monitoring in relation to preoperative ABR. This provides an idea of the prognosis. However, the most important step is to stimulate the facial nerve along the brainstem. The use of a 90-degree angled endoscope can be useful to inspect for possible tumor remnants at the fundus of the IAC. Following tumor resection, the surgical field should be copiously irrigated. Blood clots should be removed, and bleeding points should be cauterized with a bipolar electrocautery. It is important to ask the anesthesiologist to perform a Valsalva maneuver for at least 20 s to increase blood pressure. Bleeding at the CPA is the worst complication in this approach.

#### Closure

Bone edges should be carefully inspected with an angled hook. All opened air cells should be sealed with bone wax to prevent CSF leakage. A small piece of muscle or abdominal fat can be used to plug the IAC defect. A single 6.0 nylon suture can be used to hold the fat or muscle graft on the posterior wall of the IAC. Electrophysiological monitoring should be maintained at this stage for surveillance. The dural edges are reapproximated with sutures. The use of muscle tissue may be necessary to aid dural closure. Upon closure, the subarachnoid space should be copiously irrigated. The edges of the craniotomy should be inspected, and any opened air cells should be sealed with bone wax. Cranioplasty can be performed with bone cement. The scalp is closed in layers. A pressure ear dressing is applied to prevent CSF leakage.

#### Translabyrinthine

The TLB approach is indicated in patients with ipsilateral hearing loss, irrespective of tumor size. It provides excellent access to the tumor without the need for occipital or temporal lobe retraction.^153^ It also provides wide visualization of the entire facial nerve, from the brainstem to its entry into the labyrinthine portion of the Fallopian canal.^154^

Labyrinthectomy leads to complete loss of inner ear function and is therefore not suitable for patients seeking hearing preservation. Patients with VS > 2 cm located in the CPA cistern (in the extracanalicular portion) may still undergo TLB surgery, regardless of preoperative hearing. In these cases, preservation of some functional hearing is very unlikely.^155^ The presence of a high jugular bulb and a protruding (anterior) sigmoid sinus may constitute a contraindication. In these cases, skeletonization and careful removal of bone over these structures allows them to be more retractable.

### Surgical technique

#### Patient positioning

The patient is placed in supine position with the head completely rotated to the contralateral side. The surgeon may secure the patient’s head using a Mayfield clamp. The trichotomy is performed approximately 3 cm above the helix and 7–10 cm behind the postauricular sulcus. For intracanalicular tumors, the trichotomy can be performed on a smaller area of the scalp.

A curvilinear C-shaped incision is made approximately 5–6 cm behind and 3–4 cm above the postauricular sulcus. Asepsis and antisepsis of the surgical site and abdomen are performed to obtain abdominal fat for closing the dural defect. Surgical drapes should be placed on the operative site, and the abdominal region should be prepared. The incision site is injected with a 1:100,000 epinephrine and lidocaine solution.

A retroauricular C-shaped incision (arciform) is performed to elevate the musculoperiosteal flap by planes. The flap may be held in position using skin hooks or sutures. The temporal bone is exposed anteriorly to the zygoma and the External Auditory Canal (EAC), superiorly to 1–1.5 cm above the temporal line, inferiorly to the mastoid tip, and posteriorly to the region immediately after the sigmoid sinus.

#### Mastoidectomy

A wide mastoidectomy and skeletonization of the facial nerve from its vertical portion to the digastric muscle are performed. Drilling the facial recess, disarticulating the incudostapedial joint, and removing the incus allows the Eustachian tube and middle ear to be packed with small muscle fragments to avoid CSF leakage.

Bone is carefully removed from over the sigmoid sinus (up to the beginning of the internal jugular vein bulb), MF dura mater, and sinodural angle (when performing the widened TLB approach). When removing bone from over the MF dura, care must be taken to avoid injury to the superior petrosal sinus, which can lead to heavy bleeding. If bleeding occurs, pressure should be applied over a piece of absorbable hemostat with a cottonoid. Bone removal provides extra space for tumor dissection by opening the posterior fossa dura medially to the sigmoid sinus and by retracting the sigmoid sinus.

#### Labyrinthectomy

Total labyrinthectomy is performed. Drilling is first performed in the lateral semicircular canal, followed by the posterior semicircular canal and the superior semicircular canal. After total labyrinthectomy and removal of retrofacial cells, bone is removed from over the posterior and MF dura at the level of the IAC. A 180- to 270-degree skeletonization of the IAC is performed, and the IAC dura is blue-lined with care to avoid nerve injury.

The transverse crest and the vertical crest (Bill’s bar) are located at the IAC fundus, and the latter divides the facial nerve from the superior vestibular nerve. An incision is performed on the IAC dura to expose the facial, cochlear, and superior and inferior vestibular nerves. The tumor is carefully dissected and separated from the facial nerve, and its intracanalicular component is removed.

An incision is performed on the posterior fossa dura to access the tumor. Intracapsular debulking of the tumor may be performed with the use of an ultrasonic surgical aspirator or not. Dissection of the tumor capsule is then performed, carefully separating it from the facial nerve (which is positioned anteriorly in most cases), respecting the arachnoid plane. In this stage, the use of a nerve stimulator to help locate the facial nerve is recommended. Extra care must be taken when dissecting the part of the tumor attached to the brainstem. Injury to the AICA must be avoided because it carries a risk of brainstem infarction.

#### Closure

A temporal fascia or dural sealant can be used for dural closure with biological glue. The dural defect is subsequently packed with abdominal fat, which fills the space where the tumor was located in the CPA and posterior fossa, reducing the risk of postoperative CSF leak. In addition, the Eustachian tube and the middle ear may be packed with the incus and small muscle fragments to further minimize the risk of CSF leak. A titanium mesh fixed with metallic screws is placed over the layer of abdominal fat. The incision is closed in layers and tightly sutured. Drains are not typically left in retroauricular incisions but may or may not be left in abdominal incisions to prevent the formation of postoperative seromas.

#### Retrolabyrinthine approach

The Retrolabyrinthine (RL) presigmoidal transtentorial approach was described by Hitselberger in 1971 for accessing the posterior fossa and has recently been used for resection of tumors in the CPA with the aim of preserving the optic capsule.^156^ The RL approach is indicated when there is a possibility of hearing preservation. The indication depends on the level of preoperative hearing. Eligible patients must have a pure tone average > 50 dB and/or a speech discrimination score >50%, that is, must have serviceable hearing.^157^

### Surgical technique

#### Patient positioning

The patient is placed in supine position with the head turned to the contralateral side so that the biauricular plane is vertical. A C-shaped incision is performed 2 cm posterior to the retroauricular sulcus, extending from the mastoid tip to the temporal squama. The skin flap is dissected anteriorly to the cartilaginous portion of the EAC, and the digastric groove and the insertion of the sternocleidomastoid muscle are exposed at the tip of the mastoid. A large temporal fascia graft is removed from above the incision for dural closure. A U-shaped muscle flap is made to close the mastoid cavity at the end of the surgery.

#### Mastoidectomy

A canal-wall-up mastoidectomy is performed. The sinodural angle, the descending and horizontal segments of the facial nerve, the 3 semicircular canals, the sigmoid sinus, and the jugular bulb are delineated. These structures are important anatomic landmarks to obtain wider exposure of the IAC, outlining the meatal plane dura at a 180-degree angle for its exposure before opening.

A thin, depressible layer of bone may be left on the surface of the sigmoid sinus (*i.e.*, an island of bone in its central portion), which will facilitate the placement of a retractor. In addition, approximately 1 cm of posterior fossa dura is exposed with the complementary retrosigmoid drilling. This additional bone removal behind the sigmoid sinus allows retraction of the sinus posteriorly, providing improved exposure medial to the bony labyrinth of the space to be drilled, until a blue line is visible, which defines the membranous labyrinth of the posterior semicircular canal, avoiding its opening.

Drilling below the posterior semicircular canal makes it possible to reach the IAC and expose the presigmoid posterior fossa dura, which surrounds the meatal plane and should be incised just anterior to the lateral venous sinus. The retrofacial air cells are also removed, providing further exposure of the presigmoid dura (Trautman’s triangle).

#### Intracranial dissection

After dural opening, cisternal CSF partially empties into the subarachnoid space. This allows adequate visualization of the CPA with the CN VII–VIII complex emerging from the lateral pontomedullary junction, the AICA, which can be seen looping near this complex, the trigeminal nerve, the choroid plexus, the CN IX‒X complex, and the lower CNs XI and XII. The tumor may change the standard anatomic positioning by pushing the structures.

With an appropriate angulation of the microscope and retraction of the sigmoid sinus, it is possible to reach the fundus of the meatus in most cases. In those cases where this is not possible, the posterior semicircular canal is drilled and promptly occluded with bone wax. In some cases, hearing is preserved despite drilling of the labyrinth. Sometimes, a 70-degree angled endoscope is needed to inspect the fundus of the IAC for any tumor remnants.

#### Closure

The dural defect is repaired with a temporalis fascia graft and dural substitute, using fibrin glue to occlude the defect. The mastoid cavity is obliterated with a free fat graft harvested from the anterior abdominal wall in the left iliac fossa of the patient. The wound is closed in two layers: first, bone-muscle flap closure; and then subcutaneous tissue closure with absorbable 3.0 Vicryl suture and skin closure with 4.0 nylon suture.

#### Middle fossa

Microsurgical resection of VS via the MF is indicated in cases of intracanalicular tumors to preserve serviceable hearing.^149^ This approach is indicated in patients with intracanalicular VS that extend less than 1.5 cm into the CPA cistern (without contact with the brainstem), serviceable hearing (pure tone average > 50 dB and a speech discrimination score > 50%), and no contraindications to supratentorial craniotomy (age >70 years and American Society of Anesthesiologists class >2–3).^158^

Tumors that extend into the IAC fundus below the transverse crest are more difficult to remove than tumors with fundal fluid cap on the lateral aspect of the IAC.^159^ Endoscope-assisted resection has shown to be useful in the dissection of tumors that extend into the far-lateral portion of the IAC.^160^ Postoperative hearing outcomes are good in smaller tumors, particularly those smaller than 1 cm in diameter.^161^ This approach has the potential disadvantage of increased facial nerve manipulation because of its anterosuperior position in the IAC, especially for tumors arising from the inferior vestibular nerve.^162^

### Surgery

#### Positioning and exposure

The patient is placed in supine position with the head turned to the contralateral side so that the biauricular plane is vertical. The surgeon is positioned at the head of the operating table, the assistant on the right, and the microscope on the left. An incision is made in the preauricular area and extended superiorly in a curving fashion until it reaches the root of the zygomatic arch. Care must be taken near the anterior extension of the incision to avoid injury to the frontal branch of the facial nerve.

The temporalis muscle is incised beginning at the zygomatic root along the linea temporalis, elevated from the temporal fossa, and reflected anteroinferiorly. The temporalis muscle can be held anteriorly with skin hooks or silk sutures. Skin flaps are raised from the subcutaneous plan with blunt instruments such as the surgeon’s finger or the scalpel handle.

#### Intracranial access

The craniotomy is performed using cutting and diamond burs. The craniotomy measures approximately 5 × 5 cm and is two-thirds anterior and one-third posterior to the zygomatic root. The inferior limit should be at the level of the zygomatic arch, approximating the floor of the middle cranial fossa. Care must be taken to avoid laceration of the dura. The bone flap is set aside for later closure. The floor of the craniotomy should be as close as possible to zygomatic root to improve IAC exposure; it can reach as close as 1 cm from the root of the zygomatic arch. The craniotomy margins should be parallel to facilitate the positioning of the retractor on the bony borders.

The dura is elevated from the floor of the middle fossa. The first landmark is the middle meningeal artery, which represents the anterior extent of the dissection. Venous bleeding is frequently encountered in this area and can be controlled with hemostatic agents. The dura should always be elevated in a posterior-to-anterior fashion, as the geniculate ganglion is dehiscent in approximately 16% of cases. It is important to try to preserve the dural branches of the middle meningeal artery. If bleeding occurs, it can be coagulated at low power and with continuous irrigation to avoid dural laceration and future temporal lobe herniation. If dural laceration occurs, sutures can be used to fix it. Intraoperative administration of mannitol and dexamethasone is recommended to reduce intracranial pressure.

The petrous ridge is identified, and care must be taken not to lacerate the superior petrosal sinus when elevating it from the sulcus. The arcuate eminence and the Greater Superficial Petrosal Nerve (GSPN) are identified. These are the major landmarks for the subsequent intratemporal dissection. The GSPN can be found medial to the middle meningeal artery and reaches the geniculate ganglion by projecting parallel to the petrous apex. The arcuate eminence is perpendicular to the ridge. The geniculate ganglion is approximately 1 cm lateral to the petrous margin and 2 cm medial to the cortical bone. As the arcuate eminence becomes less evident, this means that the superior semicircular canal is very close. A good landmark for the IAC is the EAC, as they are well aligned.

After the dura has been elevated, the House-Urban retractor should be placed to support the temporal lobe. To maintain a secure position, the teeth of the retractor should be locked against the bone margins of the craniotomy window. The retractor blade must be placed on the inferior limit of the dissection, that is, the “true” petrous ridge. It must rest on the dura with its low extremity below the petrous ridge but without twisting the petrous sinus. Initially, the retractor is positioned without the aid of a microscope for better control of the entire operative field.

#### Internal auditory canal access

The superior semicircular canal is identified (blue-lined) at the arcuate eminence using a large diamond bur and continuous suction-irrigation. The superior semicircular canal makes a 45- to 60-degree angle with the IAC. As the arcuate eminence becomes less evident, this means that the superior semicircular canal is very close. A possible complication is superior semicircular canal dehiscence, which needs to be closed immediately with a fascia graft and bone wax or paste. Bone removal over the IAC begins medially at the porus acusticus with a large diamond bur. The area of bone anteromedial to the IAC and medial to the petrous carotid artery can expose its anterior surface. The bone in the postmeatal triangle can then be removed, exposing the posterior surface of the IAC. Medially, 270 degrees of bone can be removed from its circumference. The circumference of the IAC may be less exposed laterally because of the proximity with the inner ear. The lateral end of the IAC is dissected to expose the labyrinthine segment of the facial nerve, Bill’s bar, and the superior vestibular nerve.

The labyrinthine portion of the facial nerve is identified proximal to the ganglion. Care must be taken to avoid the cochlea, which lies less than 1 mm anterior to the labyrinthine portion of the facial nerve. This can be accomplished by careful delineation of the anterior limit of the IAC with a blunt hook. The lateral dissection is essential for clear identification of the facial nerve and complete tumor removal from the fundus of the IAC. Exposure of the meatal foramen is not recommended, as it is the narrowest portion of the fallopian canal. The dura of the IAC is divided along the posterior portion of the IAC. The facial nerve is identified in the anterior portion of the IAC. Tumor dissection begins in a medial-to-lateral direction to avoid avulsion of the cochlear nerve fibers.

The vestibular nerve is sectioned from the facial and cochlear nerves. Using a right-angled hook, the inferior vestibular nerve is divided, and the tumor is gently removed from the cochlear and facial nerves. For hearing preservation, the labyrinthine artery must be preserved. The vessel typically runs between the facial and the cochlear nerves but may not visible during the dissection. During tumor manipulation, a fenestrated neurotologic suction tip should be used to decrease suction traction on adjacent structures. Irrigation can also help clear the field and allow for gentle dissection of nerves, vessels, and the tumor.

#### Closure

After irrigation of the tumor bed and establishment of hemostasis, abdominal fat is used to close the defect in the IAC. The bone flap is repositioned. The wound is closed with absorbable sutures over a Penrose drain. This drain can be removed on the first postoperative day. A pressure dressing is maintained for 3 days postoperatively. If thinning or a tegmen tympani defect has occurred, a bone flap can be used to close the defect while keeping the bony margins intact.

#### Supportive care

Supportive care should focus on clinical symptoms and treatment complications. Trigeminal and facial nerve neuropathies, as well as brainstem compression or hydrocephalus, can be symptoms of large VSs as well as of treatment complications. Patients with facial nerve palsy may experience different types of ocular complications, such as lagophthalmos, that can lead to exposure keratopathy, corneal degradation, ulcers, and even corneal perforation. Patient management should be based on the severity of ocular symptoms and ranges from symptomatic treatment, such as eye drops, to facial nerve reconstruction via hypoglossal-facial anastomosis or oculofacial plastic surgery.^163^, ^164^ Patients with early-stage facial nerve palsy may benefit from conservative treatment; corticosteroid therapy is often recommended in these cases, but evidence for this treatment is lacking. Hearing impairment can be treated with hearing aids.^165^, ^166^

#### Quality of life

Tumor size appears to be a predictor of QoL in patients with VS. Several retrospective studies have investigated which treatment modality offers the best QoL but reached inconsistent results. Observation, resection, radiosurgery, and radiotherapy were compared using different study designs.^81^, ^84^, ^167^, ^168^ Although there are significant discrepancies between these studies, it is clear that the QoL of patients with VS cannot be predicted based on management strategy alone. Poor QoL is more likely in patients with large, symptomatic tumors that have to be removed because they pose a risk of death for the patient.^81^, ^168^, ^169^

#### Evidence of hearing preservation in different VS surgery approaches

Hearing loss is the most common symptom of VS, present in 95% of cases,^91^ and has been classified as tumorigenic or iatrogenic. It is typically sensorineural, affects the tumor ear, with slow progression, starting at high frequencies, and with reduced speech discrimination.^170^ Some patients may suffer from different degrees of sudden hearing loss that may also affect the contralateral ear.^171^

The pathophysiology of hearing loss caused by VS is still not completely understood. It probably has a multifactorial origin, and possible causes include compression of the cochlear nerve by the tumor, cochlear ischemia by occlusion of the vessels in the IAC, genetic pattern of the tumor, inflammatory mediators, and/or other secreted substances that affect the inner ear.^172^, ^173^, ^174^, ^175^

Iatrogenic causes are directly correlated with the treatment of choice. The main goal of microsurgery is total or NTR with preservation of neurological functions. During the procedure, the surgeon removes the tumor while trying to preserve the integrity of the facial and cochlear nerves.^176^ The rates of successful hearing preservation in the literature are variable, ranging from 17% to 100%. The main associated factors are tumor size, type of surgical approach, degree of preoperative hearing, and the surgeon’s experience.^177^, ^178^ The main surgical approaches aimed at preserving hearing are the MF, RS, and RL.

#### Middle fossa

The Middle Fossa (MF) approach is well known for its rates of hearing preservation, which range from 34% to 84% postoperatively. However, most studies are retrospective, and only a few prospective studies are available, but typically without comparison with other surgical techniques. The MF approach is chosen based on the surgeon’s preference, tumor size, and degree of lateral IAC involvement.^162^, ^179^, ^180^, ^181^, ^182^, ^183^

Satar et al.^179^ found that more than 60% of patients with tumors extending less than 10 mm into the CPA operated via the MF have functional hearing preservation (class A or B) and, of these, approximately 95% have good facial nerve function. Larger tumors, with 10- to 19-mm CPA extension, have lower rates of hearing preservation (34%) and facial nerve function (81%).

Wang et al.^180^ retrospectively evaluated hearing outcomes in patients with intracanalicular VS extending ≤5 mm into the IAC. Of patients with class A preoperative hearing, 67% remained class A and 17% were class B after the surgery. Of patients with class B preoperative hearing, 24% were class A and 53% remained class B after the surgery. When evaluating the durability of postoperative hearing, 65% of patients remained class A and 67% remained class B after 5 years. Given the excellent hearing results and the low surgical comorbidity, the authors suggest the possibility of intracanalicular VS resection as a first treatment option.

La Monte et al.^181^ obtained a hearing preservation rate of 58.7% (n = 37) among 63 patients undergoing MF resection of VS. Intra or extracanalicular tumor location, history of sudden hearing loss, fundal fluid cap, and tumor size were not associated with hearing preservation outcomes. DeMonte and Gidley^162^ evaluated the data of 30 patients with small tumors who underwent MF resection and found a hearing preservation rate of 73%. Arts et al.,^182^ in a retrospective study of 62 patients, also found a hearing preservation rate of 73%; when they evaluated tumors ≥10 mm, the rate decreased to 58%.

Ren et al.^183^ conducted a prospective study evaluating pre and postoperative ABR prediction of hearing preservation. They found a hearing preservation rate of 56.7%. When they evaluated tumors < 10 mm, the rate increased to 69.7% For tumors ≤5 mm and >10 mm, the rates were 84.6% and 40.7%, respectively. A stable wave V intraoperatively was a strong predictor of hearing preservation.

A few factors contribute to the good hearing results obtained with the MF approach. Dissection of the IAC exposing the medial portion first avoids injury to the inner ear and labyrinth. This approach also provides enhanced lateral exposure of the IAC, allowing complete resection of the tumor in a better dissection plane and with less traction on the cochlear nerve. The cochlear nerve is weaker on the distal portion, where it forms fine filaments and is more susceptible to inadvertent surgical manipulation. Intraneural trauma and changes in blood supply are two possible causes of postoperative hearing loss when the cochlear nerve remains intact.^184^

The MF approach is the preferred choice for many surgeons, especially neurotologists, when operating small tumors with limited APC extension. For larger tumors with greater APC extension that compress or are in contact with the brainstem, the preferred approach is the RS.^185^ As tumor size is the only unanimous factor associated with postoperative hearing outcomes, the fact that the MF approach is more used in small tumors could explain the good postoperative results. However, studies without selection bias showed similar rates of hearing preservation between surgical techniques.^178^

#### Retrosigmoid

The RS approach has similar hearing preservation rates to the MF approach, especially when performed in small or intracanalicular tumors.^89^, ^183^, ^186^ Bozhkov et al.^89^ retrospectively evaluated 138 patients undergoing RS resection of VS. The overall hearing preservation rate was 32%. However, for tumors < 12 mm, hearing preservation was achieved in 83.3% of patients. For intracanalicular tumors, the preservation rate was 100%, whereas only 5.3% of patients with tumors >25 mm had preserved hearing. Ren et al.^183^ found a hearing preservation rate of 41.7% (63 out of 151 patients). This rate was higher for intracanalicular tumors than for tumors with an APC component (57.6% vs. 29.4%, *p* = 0.03).

Preet et al.^177^ conducted a systematic review of hearing preservation rates in patients who underwent RS resection of VS. The hearing preservation rate ranged from 31% to 35%. Patients were stratified according to the degree of cisternal involvement into three groups: intracanalicular, small (0–20 mm), and large tumors (≥20 mm). Hearing preservation rates according to tumor size were 48%, 35%, and 12%, respectively. Intracanalicular tumors were associated with a higher rate of hearing preservation when compared with small and large tumors (*p* < 0.04).

Yates et al.^186^ retrospectively evaluated the rate of hearing preservation in patients with large tumors undergoing RS resection. Sixty-three patients with class A or B hearing and an APC component ≥ 15 mm were included. Overall, only 4 patients maintained good postoperative hearing (6.3%). The group with smaller tumors (between 15 mm and 19 mm) had a higher rate of hearing preservation (17.6%; 3 out of 17 patients). No patient with an APC component >25 mm had their hearing preserved after surgery (0 of 23 patients).

Tumors with large extracanalicular components are associated with limited hearing outcomes, with a low rate of preservation. The great mass effect and the need for mobilization, especially proximally and distally to the auditory nerve, can damage the cochlear nerve or the cochlear nuclei. In addition, tumor compression of the nerve is increased, as well as tumor adhesion to neurovascular structures. This makes tumor resection more complex, with reduced chances of preserving the labyrinthine artery and the integrity of the cochlear nerve.^177^

#### Retrolabyrinthine

Another alternative for accessing the IAC and the posterior fossa for VS resection with the possibility of hearing preservation is the RL approach. It was first described by Hitselberger and Pulec in 1971 for sectioning the trigeminal nerve for the treatment of neuralgia^156^ and only later used for VS resection. Advantages are the same as the TLB approach, but with the possibility of hearing preservation.^157^, ^187^, ^188^, ^189^

Bento et al.^187^ used this approach for VS resection and were able to achieve facial nerve and hearing preservation, with no morbidity. Of the 22 patients who underwent surgery, 31.8% maintained similar hearing levels as preoperatively. The fundus of the IAC could not be reached in 3 patients, requiring the use of the TLB approach. Surgery was most challenging in patients with a high jugular bulb or limited space between the sigmoid sinus and the Posterior Semicircular Canal (PSC).

Darrouzet et al.^189^ used a modified RL approach to treat 60 patients with VS. The widened RL approach consists of exposing the MF and RS dura mater to achieve enhanced visualization of the IAC to allow resection of larger tumors with deeper penetration into the IAC fundus. The overall rate of hearing preservation was 21.7%, but 30% of patients had a tumor larger than 25 mm. In a subgroup of 20 patients with good preoperative hearing and an intracanalicular tumor involving less than half of the IAC, the hearing preservation rate was 50%.

Bento and Lopes^157^ retrospectively evaluated 189 patients who underwent RL resection of VS. Audiologic evaluation at 90 days after surgery revealed that 94 patients (49.73%) had the same level of hearing as preoperatively (class A or B). Wang et al.^188^ evaluated the surgical results of 10 patients and reported a hearing preservation rate of 50%. Sass et al.^190^ evaluated 31 patients and reported a hearing preservation rate of approximately 77% after extended RL VS surgery using a new monitoring system in which the electrode is placed on the foramen of Luschka.

Although the hearing preservation rates of RL VS surgery are similar to those achieved using the other two surgical techniques, there are only a few studies using this approach, and most of them are retrospective. There are no prospective randomized studies comparing the RL approach with the other approaches in terms of postoperative hearing preservation. The low morbidity and mortality, in addition to preservation rates similar to those of other techniques, should lead to the widespread use of this technique.

#### Middle fossa vs retrosigmoid

Published studies comparing the RS and MF approaches in terms of hearing preservation are scarce. Most of them are retrospective, and the choice of surgical approach is often based on the surgeon’s preference and tumor size.^78^, ^97^, ^147^, ^184^^,^^191^ In general, statistical analyses do not favor any approach, except when involving intracanalicular tumors, for which some studies suggest that hearing preservation is more commonly achieved with MF resection (Table 11).^97^, ^184^

**Table 11:** Hearing preservation rates of studies comparing Middle Fossa (MF) vs. Retrosigmoid (RS) resection of vestibular schwannoma.

Authors	Patients (n)	MF	RS
Irving et al.^20^	98	52%	14%
Rabelo de Freitas et al.^21^	176	47.8%	23.5%
Hillman et al.^22^	138	59.3%	38.5%
Sameshima et al.^23^,^a^	125	76.7%	73.2%
Wilkinson et al.^24^	377	71%	59.3%
Colletti and Fiorino^27^,^b^	50	52%	40%

a Only tumors smaller than 15 mm.

b Only intracanalicular tumors.

Irving et al.^184^ retrospectively compared MF vs. RS resection of VS. Preservation of serviceable hearing was achieved in 52% of patients who underwent MF surgery, whereas only 14% of patients who underwent RS surgery had preserved hearing. For intracanalicular tumors and tumors with a CPA component measuring between 1 and 10 mm, the FM approach was superior to RS in preserving hearing (*p* = 0.009 and *p* = 0.006, respectively).

Rabelo de Freitas et al.^147^ evaluated 176 patients and found that hearing preservation was more likely when using the MF approach instead of the RS approach (48.8% vs. 23.5%, respectively; *p* = 0.0008), especially for intracanalicular tumors. Hillman et al.^97^ conducted a similar study and also found increased rates of hearing preservation in patients undergoing MF surgery compared with those undergoing RS surgery (59.3% vs. 38.5%, respectively; *p* = 0.02).

Sameshima et al.,^78^ when evaluating hearing preservation in patients with VSs smaller than 15 mm, found similar results between the MF and RS approaches (76.7% vs. 73.2%, respectively; *p* = 0.9024). However, the MF approach had a higher rate of complications. Wilkinson et al.,^191^ in a larger study population, found that patients undergoing RS surgery were more likely to experience hearing loss than patients undergoing MF surgery, irrespective of tumor size. There were no differences in comorbidity and complication rates between the two approaches, but preservation of facial nerve function (House-Brackmann [HB] I) was higher in the RS group (p ≤ 0.004).

Colletti and Fiorino^192^ conducted a prospective randomized study comparing the MF and RS approaches. In general, there were no significant differences between the two approaches, but MF surgery had better results than RS surgery when the tumor reached the IAC fundus (60% vs.44%, respectively). Exposure of the IAC fundus is limited when using the RS approach, as it provides access to only 80% of the IAC. In addition, the closer the tumor to the IAC fundus, the higher the probability of PSC injury or direct cochlear nerve injury. In MF surgery, complete exposure of the IAC is performed extradurally. The risk of inner ear injury is lower, and tumors smaller than 15 mm are more easily accessed through this technique.

There are no well-designed published studies that prove the effectiveness of one surgical technique over the other in terms of hearing preservation after VS resection. Most studies are retrospective case series, and although there are a few prospective studies, none of them are randomized. The lack of data standardization also hinders a comparative analysis of techniques and different studies. In general, hearing preservation rates are similar between the different surgical approaches, with a greater probability of success when the patient has intracanalicular tumors or tumors with little APC extension.

#### Recommendations

XVI – There is not sufficient evidence to favor one surgical approach over the other,^193^ therefore no recommendation can be made. The approach should be chosen individually in each case, according to tumor characteristics and clinical aspects.
XVII – Patients with serviceable hearing may undergo RS, MF, or RL resection of VS. Strong recommendation. Moderate degree of evidence.
XVIII – The MF approach has higher rates of hearing preservation than the RS approach for Koos grade I and II. Moderate recommendation. Low degree of evidence.

### Radiosurgery and radiotherapy

SRT is a noninvasive technique that provides high doses of irradiation to small volumes of target tissue. Its use for the treatment of facial nerve neuromas was first described in 1971 by Leksell.^194^ Currently, SRT is recommended for tumors that do not exceed 3 cm in diameter.^195^ Some studies report good clinical outcomes with good tumor control, low rates of facial nerve involvement, and relatively low costs.^134^, ^196^, ^197^, ^198^, ^199^ However, most studies reporting a lack of tumor growth after treatment did not assess whether the tumor was growing before SRT.^199^

SRT is divided into four methods: SRS, Fractionated SRT (FSRT), Conventional SRT (CSRT), and proton beam therapy.^32^ The choice of a particular method is based on the preference of the attending physician and the patient, tumor size, clinical condition, and the degree of hearing impairment or preservation presented by the patient. Similar rates of successful treatment have been reported for all methods when comparing similar tumors.^198^

Tumor size plays a major role in the final decision regarding the radiotherapy method to be used. SRS is considered for tumors up to 2.5 cm in extracanalicular diameter, and SRT for tumors up to 3.5 cm. Patients with larger tumors may also undergo radiotherapy^200^ All these methods have the goal of arresting tumor growth while preserving adjacent structures. STR is not recommended for larger tumors with symptoms secondary to mass effect or brainstem compression.^32^

#### Stereotactic radiosurgery

SRS delivers a single conformal high dose of radiation and is commonly used in small or medium VSs. Radiosurgery may be performed with a cobalt-60 source GammaKnife or Linear Accelerator (LINEAC) techniques such as the CyberKnife at doses of 11–14 Gy.^134^, ^167^, ^201^ Theoretically, it minimizes injury to adjacent structures such as the trigeminal and facial nerves. These methods require spherical targets with a diameter less than 3 cm.^93^

Early treatments used high doses of radiation that led to good tumor control rates but significantly affected adjacent structures, with a medium-term hearing preservation rate of 40% and a facial paralysis rate of 33%. Technological advances allowed the use of lower radiation doses that still arrest tumor growth while producing acceptable rates of side effects and a rate of facial nerve injury of only 5%.^202^ Hearing preservation rates, even with lower radiation doses, are reported to be approximately 25%.^203^, ^204^, ^205^

Several retrospective cohort studies have evaluated SRS using GammaKnife with at least 100 patients, 2 years of follow-up, and objective audiometric evaluation. A series of more than 100 patients undergoing LINEAC surgery with a 2-year follow-up period did not report hearing function outcomes.^206^ The pioneering SRS series included patients treated with very high-dose regimens. A contemporary SRS series using GammaKnife with tumor margin doses of 12–14 Gy revealed 5 year tumor control rates of 90%–99%, hearing preservation rates of 41%–79%, facial nerve preservation rates of 95%–100%, and trigeminal nerve preservation rates of 79%–99%.

Several studies have found that the main predictor of hearing preservation after radiosurgery is the degree of preoperative hearing.^207^, ^208^, ^209^, ^210^ A recent review reported significant hearing deterioration after SRS, even in patients with normal hearing function (Gardner–Robertson grade 1).^132^ The probability of hearing preservation was >75%–100% after 2 years, >50–75% after 5 years, and >25%–50% after 10 years. The rates of hearing preservation after 5 and 10 years were similar to those of patients undergoing microsurgery.^132^ However, it should be noted that these data are based on patients who underwent surgery with the specific goal of preserving hearing.

The maximum dose to the modiolus of the cochlea has been reported as a negative predictor of functional hearing preservation, with a threshold of approximately 4 Gy.^208^, ^211^, ^212^, ^213^ However, these series consist of small retrospective patient cohorts. The cochlear dose is likely to be one of many variables associated with hearing preservation. SRS doses of 11–14 Gy are recommended for tumor margins and doses of 11–12 Gy are recommended when the risk of hearing loss is critical.^214^

After treatment, additional radiotherapy may be required, or the patient may become eligible for conventional surgery. The higher the rate of tumor growth before the intervention, the higher the rate of reintervention.^146^

#### Fractionated stereotactic radiotherapy

FSRT delivers focused doses of radiation given over a series of radiotherapy sessions. It aims to control tumor growth with the least possible injury to surrounding neural structures, especially the facial nerve. Although fractionated radiation doses have no radiobiological benefit, FSRT allows a more homogeneous and less conformal dose deposition, especially in irregularly shaped tumors.^32^

One of the largest series of patients (n = 383) treated with FSRT with long-term follow-up reported a tumor control rate of 96% at 5 years and a serviceable hearing preservation rate of 76%, with no cases of facial nerve dysfunction, at 3 years.^215^ Conversely, a systematic review comparing SRS and FSRT reported similar tumor control rates between the two modalities, but a lower risk of medium- and long-term facial and trigeminal nerve deterioration in patients treated with SRS. The review also reported similar rates of hearing preservation between the two techniques.^216^ These results are inconclusive. Quality studies are lacking.

#### Proton beam therapy

Proton beam therapy delivers a high energy proton beam to the target tissue, damaging the DNA of the targeted cells. Damage to the tumor is caused not only by direct collision with protons, but also by indirect local damage caused by oxygen radicals released during the process. Selected protons have a predetermined range, meaning that very few can penetrate beyond the pre-established tumor limits, with the dose delivered being maximized over the last few millimeters of the protons’ range (known as the Bragg peak). This feature minimizes damage to surrounding healthy tissue.^217^

There are only a few published studies evaluating proton beam therapy for VS treatment. A tumor control rate of 93.6% has been reported, as well as a 67% rate of irreversible audiologic trauma. The rates of facial and trigeminal nerve injury were 8.8% and 10.6%, respectively.^217^ The technical, logistical, and financial difficulties of proton beam therapy prevent further studies from being conducted, and this technique still lacks greater scientific validation regarding doses and long-term follow-up. In addition, the associated high costs make this modality unfeasible in many countries.

#### Radiosurgery

Four nonrandomized studies compared the results of observation vs. SRS and found better tumor control rates after treatment with SRS (class of evidence II; level of recommendation B).^97^, ^218^, ^219^, ^220^ Some studies reported lower rates of hearing loss in patients who underwent SRS, while others reported similar hearing outcomes and complaints between the two modalities.^97^, ^218^, ^219^, ^220^ Two studies compared conservative management, surgery, and SRS using several QoL questionnaires after 5–7 years of follow-up.^81^, ^221^ Both reports showed that patients undergoing conservative management only responded more favorably than those who were treated early. In addition, patients undergoing observation had better hearing and facial nerve outcomes, the latter only in relation to surgery. It should be noted that in almost all patients undergoing observation, tumor size remained stable, which although may represent a relevant bias, also indicates the importance of treatment weighting.

#### Recommendations

There is little information on the incidence of malignant VS after radiation of sporadic, non-NF2 VS. The risk of spontaneous malignancy was addressed in a large retrospective study with data from the SEER database. The incidence of MPNSTs in CN VIII in patients without history of previous radiation was 0.017 per 1 million person-years. Compared with the incidence of VS, 1041 VS were present for every 1 MPNST arising from CN VIII. There is no evidence that MPNST is an exclusive feature of NF2 in relation to NF1; however, approximately half of MPNSTs reported after radiotherapy occurred in patients with NF2.^222^ This baseline malignancy rate should be considered when estimating the risk of malignant transformation after SRS for VS.^223^ In a single-center retrospective review, Pollock et al.^224^ did not identify radiation-induced tumors in 11,264 patient-years of follow-up after SRS. In a review by Maducdoc et al.,^225^ there were only 8 patients with malignant transformation after surgery or SRS, of whom 4 only underwent surgery.

Several studies have reported on transient VS growth occurring within 3 years after radiosurgery.^226^, ^227^, ^228^ This MRI alteration observed in up to 30% of patients is related to the therapeutic effect of SRS and is called “pseudoprogression”. It is not a predictor of treatment failure.^228^, ^229^

SRT is an option for the management of patients with VS. However, there is no high-quality evidence in the literature to support that this treatment modality is preferable to the “wait and watch” approach or to classic resection. Four nonrandomized studies compared the results of observation vs SRS and found better tumor control rates after SRS.^97^, ^218^, ^219^, ^220^ Although some studies have reported lower rates of hearing loss in patients who underwent SRS, others found no significant differences in hearing outcomes and complaints.^97^, ^218^, ^219^, ^220^ The rates of hearing preservation are still poor.

The major limitation of evaluated studies is the lack of patient randomization, as randomization in the follow-up of conditions such as VS has relevant ethical issues. In addition, the unavailability of radiotherapy in many centers makes it difficult to standardize applied therapies and data collection. It should be noted that few studies considered late malignant transformation as a possible therapeutic effect of radiotherapy.

XIX – Radiotherapy may be indicated as primary treatment in selected cases; however, there is still controversy surrounding long-term effectiveness and safety, especially in young patients. Moderate recommendation. Low degree of evidence.
XX – Radiotherapy may be indicated in patients with clinical contraindications (comorbidities) to surgery. Strong recommendation. Moderate degree of evidence.
XXI – Radiotherapy is not routinely indicated in patients with NF2. Moderate recommendation. Moderate degree of evidence.

### Systemic treatment

Research on genes involved in NF2 provide a good pharmacological basis for the development of targeted VS therapy. Compared with traditional chemotherapy, targeted therapy causes less nerve and vascular damage. Mutation of the NF2 gene and consequent dysfunction of its transcription product, merlin (moesin-ezrinradixin-like protein), are typical features of NF2-related schwannomas and frequently occur in sporadic VS.^31^

Schwann cells remain quiescent because merlin regulates contact-dependent inhibition of proliferation. Alterations in merlin cause activation of pathways related to cell growth and proliferation. Approximately 60% of unilateral and 90% of bilateral VS (NF2) cases have an NF2 gene mutation and alterations in the merlin transcript.^31^, ^230^

The understanding of the mechanisms by which merlin dysregulation induces tumor growth, as well as of signal pathways related to VS growth, has raised hopes for the application of targeted therapies. Merlin plays a key role in regulating actin cytoskeleton-mediated processes, cell proliferation, and adherens junction formation.^231^ As it can regulate multiple pathways involved in tumor genesis, all these sites would be potential therapeutic targets for VS.^232^, ^233^, ^234^

Some of these pathways include Retrovirus-Associated DNA Sequences (Ras), Rapidly Accelerated Fibrosarcoma (Raf), Mitogen Extracellular signal-regulated Kinase (MEK), Extracellular-signal-Regulated Kinases (ERK), mammalian Target of Rapamycin Complex 1 (mTORC1), Rac, p21-Activated Kinase (PAK), C-Jun kinase, Phosphoinositide 3-Kinase (PI3K), Akt, and the intranuclear E3 ubiquitin ligase CRL4 (DCAF1).^235^

SH3PXD2A-HTRA1 fusion has also been suggested as a possible mechanism of VS genesis. The product of this fusion leads to tissue proliferation and invasion. A previous study found this fusion in 10% of patients with sporadic VS.^23^ Another study showed that this transcript is quite rare.^236^ Although the biomechanical consequences of this fusion need further elucidation, activation of the MEK-ERK pathway seems to be involved.^23^ Further studies are needed to assess the importance of this fusion and potential therapeutic options.

#### Protein tyrosine kinase inhibitor

Kinases catalyze the transfer of phosphate on Adenosine Triphosphate (ATP) to determined target proteins, thereby regulating cell differentiation, growth, migration, and apoptosis. Protein Tyrosine Kinases (PTKs) catalyze the transfer of phosphate to tyrosines and can be divided into Receptor protein Tyrosine Kinases (RTKs) and nonreceptor Protein Tyrosine Kinases (nrPTKs) according to their structure.^23^ RTKs such as ErbB, Platelet-Derived Growth Factor (PDGF), Fibroblasts Growth Factor (FGF), insulin-like growth factor 1, and Vascular Endothelial Growth Factor (VEGF) have been shown to be associated with VS.^235^, ^237^, ^238^, ^239^, ^240^ The activation of nrPTKs, which was also found in NF2, also promotes cell proliferation, cell apoptosis resistance, and oncogenesis.^241^

#### Vascular endothelial growth factor receptor inhibitors

VEGF Receptors (VEGFRs) are important regulators of angiogenesis, and merlin mutations can increase VEGFR-mediated angiogenesis.^242^, ^243^ This suggests that the anti-VEGF monoclonal antibody bevacizumab may have a role in VS treatment. This is the most studied drug class in the clinical treatment of VS. A prospective, multicenter, uncontrolled phase II study of 14 patients with progressive NF2-related VS revealed hearing improvement in 36% of them, with no cases of hearing deterioration within 12 months.^244^

Plotkin et al.^245^ reported that bevacizumab led to tumor shrinkage in more than 90% of patients with progressive NF2-related VS, as well as hearing preservation in more than half. Another study by Plotkin et al.^246^ showed tumor regression in 40% and hearing improvement in 20% of patients with progressive NF2-related VS. A systematic review evaluating the safety and efficacy of bevacizumab reported tumor regression in 41% of patients, as well as hearing improvement in 20% and hearing stability in 69%. These data supported the decision of the UK National Health System to fund the use of bevacizumab. Data were based only on patients with NF2.^247^

Bevacizumab can be considered a first-line treatment for fast-growing VS. The first phase III clinical trial of bevacizumab was conducted in 2021 in Japan.^248^ However, the use of bevacizumab has some disadvantages such as parenteral administration, side effects, drug resistance, and rebound tumor growth.^249^ Most published case series draw conclusions on treatment efficacy based on relatively short follow-up periods. Long-term follow-up studies with a larger patient population are needed. The efficacy of bevacizumab in the pediatric population is controversial. One study showed significant tumor reduction after 1 year of treatment,^250^ whereas others showed minor radiologic responses despite a postponement in hearing loss.^246^, ^251^ It has poor efficacy in small, slow-growing tumors and after partial resection.^252^

Treatment with bevacizumab is longer in VS when compared with malignant tumors. A meta-analysis reported a mean treatment period of 16 months.^253^ In a retrospective study of 33 patients with NF2, 58% developed hypertension and 62% developed proteinuria, requiring dose adjustments.^254^ Therefore, a low-dose regimen may be more appropriate in prolonged treatments.^255^ Selective intra-arterial use can also increase drug concentration in the tumor, with a better therapeutic effect. The role of bevacizumab is best documented in NF2. Karajannis et al.^256^ reported a case of tumor shrieking in a patient with sporadic VS.

A clinical trial of VEGFRs 1/2 peptide vaccination in patients with NF2 showed hearing improvement and tumor volume reduction. This was the first immunotherapy approach for patients with NF2. The safety and preliminary efficacy of VEGFRs peptide vaccination in patients with NF2 raised hopes for NF2-related VS immunotherapy.^257^

#### ErbB protein inhibitors

When ErbB family’s cell membrane receptors (ErbB-2 and Epidermal Growth Factor Receptor [EGFR] in VS) are activated, they form heterodimers and activate intracellular kinases. Activation of ErbB receptors is a common feature of sporadic and NF2-related VS and is associated with EGFR expression. EGFR levels are directly correlated with VS tumor size and inversely with patient age. In addition, EGF is upregulated in NF2-related VS but not in sporadic VS. Therefore, EGFR inhibitors may be effective in patients with NF2.^258^, ^259^

The anti-ErbB2 monoclonal antibody trastuzumab reduces VS cell proliferation but does not significantly increase cell death.^260^ Lapatinib is a potent and reversible tyrosine kinase inhibitor that has been widely used in the treatment of metastatic breast cancer. It has an inhibitory effect on EGF activation and, therefore, may be effective in stopping the growth effects of EGF in VS. A phase II study showed that lapatinib has low toxicity but poor effect on reducing tumor volume and improving hearing in patients with progressive NF2-related VS.^261^

Erlotinib, a reversible EGFR-specific tyrosine kinase inhibitor, was able to reduce VS growth in mice but failed to reduce tumor growth and improve hearing in 11 patients with NF2. Bevacizumab proved to be more effective in treating patients with NF2 than lapatinib and erlotinib.^262^

#### PDGFR inhibitors

Platelet-Derived Growth Factor receptors (PDGFs) regulate the migration of mesenchymal stem cells. Compared with normal nerves, the expression and activation of PDGFR family proteins are increased in sporadic and NF2-related VS. Therefore, they are candidates as VS therapeutic targets. *In vitro* studies showed that imatinib mesylate increases apoptosis, decreases cell viability, and inhibits angiogenesis.^263^, ^264^

Nilotinib, a second-generation RTK inhibitor, has a 10–30 times greater *in vitro* effect than imatinib and greater blood-brain permeability. Its antitumorigenic effect is related to the inhibition of PDGFRs and their downstream signaling pathways, Akt and mTOR.^265^ Ponatinib, a third generation PDGFR inhibitor, inhibits fibroblast growth factor receptor, PDGFR, and VEGFR. It can reduce the viability of VS cells with NF2 mutations and might also have a therapeutic effect in VS. However, it has not shown significant clinical benefits in patients with NF2.^266^

#### HGFR inhibitors

The activation of Hepatocyte Growth Factor Receptor (HGFR), also known as c-mesenchymal-epithelial transition, in sporadic VS can promote tumor growth,^267^ as it mobilizes the inflammation network and consequently leads to cancer progression.^268^ It is also related to chemotherapy and radiotherapy resistance through the PI3K/Akt signaling pathways, reducing the apoptosis induced by these treatments.^269^, ^270^ The HGFR inhibitor crizotinib can enhance cellular radiosensitivity in NF2, allowing reduced doses of radiotherapy and thereby protecting hearing.^271^ A phase II clinical trial of crizotinib for NF2-related and sporadic VS in children and adults is ongoing (NCT04283669).^235^ The combined use of the c-MET inhibitor cabozantinib and the Src inhibitor saracatinib is more effective in reducing the viability of VS cells with a NF2 mutation than using either drug alone.^272^

#### Small molecule inhibitors of Akt signal transduction

The PI3K/Akt pathway is the most studied abnormality in VS and is manifested by elevated mRNA and protein levels.^173^ This pathway is a confluence point of many cell proliferation and differentiation processes, contributing to tumorigenesis. Therefore, it is an attractive therapeutic target for VS treatment.^273^ *In vitro* and animal studies show that PI3K/Akt pathway inhibitors such as OSU-03012 (AR-12) and OSU-HDAC42 (AR-42) inhibit cell growth and induce apoptosis in VS cells, with a promising therapeutic potential.^274^, ^275^

#### mTORC1 inhibitors

mTOR, a signal of the PI3K/Akt pathway, integrates multiple intracellular environments, working as a central hub in these cascades. As merlin reduces mTORC expression, mTORC1 inhibition in merlin-deficient tumors may be a therapeutic target.^232^ The mTOR inhibitor rapamycin (sirolimus) inhibited NF2-related VS cell growth *in vitro* and may lead to tumor shrinkage in patients with NF2 with growing tumors *in vivo*.^276^ Everolimus, a derivative of rapamycin, can inhibit mTORC1 and angiogenesis. It was ineffective in patients with NF2-related VS in a phase II study, but reduced tumor growth by 55% in patients with NF2 in another study.^277^ The effect of everolimus is still under discussion.

#### Cytokines and chemokines

##### Chemokine receptor type 4 inhibitors

Chemokine Receptor Type 4 (CXCR4) is a protein that has an important role in neural development and pathological processes such as tumor development. It also plays a role in angiogenesis, metastasis, and in the tumor microenvironment. Furthermore, this cytokine produces chemoresistance and is related to the genesis of sporadic NF2-related VS.^278^

CXCR4-directed Positron Emission Tomography/CT (PET/CT) was used to evaluate CXCR4 expression in patients with VS. Its detection in tumors with the use of specific markers in PET/CT may allow the use of CXCR4 inhibitors (such as plerixafor) on an individual basis.^278^

#### Inflammatory factor inhibitors

##### Cyclooxygenase 2 inhibitor

Cyclooxygenase 2 (COX2) expression is associated with sporadic and NF2-related VS. Prostaglandin E2 catalyzed by COX2 has a role in cell proliferation, angiogenesis, and inflammation. This means that COX2 inhibition may have the potential to inhibit VS growth. A negative correlation between aspirin users and sporadic VS growth has been found, indicating a potential therapeutic role of aspirin.^279^ However, celecoxib and aspirin did not inhibit sporadic and NF2-related VS growth in other studies, although tumor growth was not observed. Other studies showed that corticosteroids, nonsteroidal anti-inflammatory drugs, and other immunosuppressive drugs did not alter COX2 expression in sporadic VS.^280^, ^281^

The use of aspirin was recommended in “wait and scan” patients with VS to prevent tumor proliferation.^282^ A meta-analysis published after this recommendation showed no difference in tumor growth between patients using and not using aspirin, suggesting that there is insufficient evidence to recommend aspirin in VS. Randomized clinical trials are needed to determine the effectiveness of aspirin on schwannoma growth.^283^

##### NFκ inhibitor

Mifepristone, a progesterone, and glucocorticoid receptor antagonist is currently used for medical abortion and is considered a promising drug in VS treatment. It acted on inflammatory markers of VS and inhibited proliferation of VS cells in *in vitro* studies, irrespective of the presence of an NF2 mutation. A phase II clinical trial is being programmed.^284^

#### Tumor microenvironment

Schwannomas consist of different cell types, which include tumorigenic Schwann cells, axons, macrophages, T-cells, fibroblasts, blood vessels, and an extracellular matrix. The tumor microenvironment plays a relevant role in the development and progression of schwannomas. There are only a few studies on the tumor microenvironment of schwannomas.^285^

Fast-growing tumors are associated with the expression of factors such as M-CSF and IL-34, which regulate Tumor-Associated Macrophages (TAMs). TAMs inhibit the immune response, resulting in tumor progression. Other examples of microenvironment alterations are the expression of programmed cell death 1 expressed on CD8+T-cells and regulatory T-cells (CD4+ CD25+ Foxp3+).^286^

Hypoxia may be important in shorter progression-free survival in NF2. Immunotherapy targeting the tumor microenvironment may emerge as a new class in the treatment of shwannomas.^287^ In addition to those mentioned previously, the action of other drugs is also being studied to support individualized treatment, especially in NF2. The complex interconnected pathways in VS genesis suggest that combination therapy may offer the optimal therapeutic effect.

#### Recommendations

Of all drugs with a potential role in VS treatment, bevacizumab showed the greatest progress and best results. It was recently considered a first-line treatment for fast-growing VS. In addition, recent therapeutic strategies targeting the SH3PXD2A-HTRA1 fusion, several protein kinases, and the tumor microenvironment may further support VS management. Immunotherapy may be necessary to control multiple tumor progression in the long-term.

Despite advances in targeted therapy, potential toxicity and side effects need to be considered given the prolonged period of VS treatment. High-level evidence to support systemic VS treatment is lacking. Thus, systemic treatment options are typically used in patients who underwent previous local therapy, which potentially limits and interferes with the ability to determine drug effectiveness.

XXII – Aspirin use in patients with sporadic or NF2-related VS. Not recommended. Insufficient evidence.
XXIII – Use of EGFR inhibitors (erlotinib and lapatinib) and the mTOR inhibitor everolimus in sporadic or NF2-related VS. Not recommended. Insufficient evidence.
XXIV – Bevacizumab use in sporadic VS. Not recommended. Insufficient evidence.
XXV – Bevacizumab is a treatment option for patients with NF2 and could be prescribed for patients with fast-growing VS and/or progressive bilateral sensorineural hearing loss. Moderate recommendation. Low level of evidence.

Local therapy is the basis of VS treatment. There is no level I evidence for any systemic treatment, and even level II evidence for treatment with the antivascular EGF antibody bevacizumab is debatable and valid only for NF2-related VS. Among available therapies, safe surgical resection and radiotherapy are considered superior. Thus, systemic treatment options are typically used in patients who underwent previous local therapy, which potentially limits and interferes with the ability to determine drug effectiveness.

### Evidence of hearing preservation in different VS approaches

Treatment modalities have tried to maintain satisfactory hearing levels for communication in patients.^288^ Published studies report their hearing results in different ways, following different classifications of audiologic patterns and hearing preservation. The most used classification is from the AAO-HNS Foundation.^54^ Some reports use the classifications by Gardner and Robertson^131^ and Meyer et al.^289^

#### Wait and scan

The “wait and scan” approach is recommended in patients with small tumors and a low rate of annual growth.^288^ Older patients and those with comorbidities that preclude active treatment are eligible for this modality. In 2019, Reznitsky and Cayé-Thomasen^290^ conducted a systematic review of 15 cases series including 2142 observed patients and found a hearing preservation rate of 54% after 5 years of observation.

#### Radiotherapy

There are many reports of hearing preservation after different modalities of radiotherapy for VS, especially in the past 2 decades.^291^ Hearing preservation rates of more than 40% have been reported after 10–15 years.^292^, ^293^, ^294^ Some studies^204^, ^205^, ^295^ reported hearing deterioration after long-term follow-up in patients treated with radiotherapy, including those who underwent LINEAC techniques. Carlson et al.^205^ reported a hearing deterioration rate of 60% in patients with 1-cm tumors after 4.2 years of follow-up. Only 23% of patients maintained serviceable hearing at 10 years. Hasegawa et al.^204^ found hearing preservation rates of 64% for patients with class I hearing and of 24% for patients with class II, suggesting that pretreatment hearing is an important prognostic factor for hearing preservation after radiotherapy.

Coughlin et al.^295^ conducted a systematic review to estimate hearing preservation rates at 10 years after GammaKnife radiosurgery. They revealed a significant reduction in the percentage of patients who maintained serviceable hearing over the years. Interaraural correction showed, in the contralateral ear, an 8-dB mean decrease in PTA and a 1.8% decrease in word recognition scores per year. Different radiotherapy modalities did not prove to be effective for serviceable hearing preservation in the 10-year follow-up period.

#### Surgery

Surgery has been the best option for serviceable hearing preservation in patients with VS, but several factors should be considered, such as position of the tumor in the IAC, tumor size, and audiologic assessment at diagnosis. The choice of surgical approach (MF, RS, or RL) depends on the surgical team’s experience. Carlson et al. investigated the variables directly affecting maintenance of serviceable hearing. Patients with AAO-HNS class B hearing were 2.4 times more likely to lose serviceable hearing and become class C or D than patients with class A.^205^

A retrospective analysis^296^ of 152 patients operated via the MF showed a preservation rate of 62%, with 52% within 15 dB and 15% discrimination. After dividing patients according to tumor size, Wilkinson et al.^191^ found that hearing preservation was greater in patients undergoing MF surgery instead of RS.

Evidence of surgery indication is based on studies showing that the main predictive factors for hearing preservation are tumor size and preoperative hearing. Stangerup et al.^297^ found that small hearing impairments lead to significantly lower proportions of hearing preservation and maintenance. According to Brackmann et al.,^116^ normal preoperative hearing on the affected side, reduction of wave V latency, and presence of CSF at the IAC fundus are associated with hearing preservation.

#### Recommendations

XXVI – Patients with intracanalicular tumors and normal hearing could undergo to “wait and scan protocol”, but if there is evidence of tumor growth and/or deterioration of hearing function, and/or vestibular symptoms refractory to clinical treatment, surgical intervention could be performed. Strong recommendation, low degree of evidence.
XXVII – Patients with serviceable hearing should not undergo radiotherapy as a first treatment option in order to preserve hearing, except when there are clinical contraindications to perform surgery. Strong recommendation, moderate degree of evidence.
XXVIII – Surgery is superior to radiotherapy in order to preserve serviceable hearing in patients with VS. Strong recommendation, moderate degree of evidence.

Preservation of facial nerve integrity is now possible in most cases and is directly associated with tumor size. More refined surgical techniques, with the advent of more appropriate instruments, intraoperative monitoring of facial and auditory nerves, and ultrasonic aspirators, have made surgery much safer.

Preservation of facial nerve function is considered successful when patients achieve HB I or II postoperatively, even if there is a transient worsening in the immediate postoperative period. In a Brazilian study of 825 patients undergoing MF, RL, TLB, or combined surgery, 85.6% had preserved facial nerve function (HB I or II).^298^

When comparing different surgical approaches, considering tumor size in relation to preservation of facial nerve function, Ansari et al.^299^ showed that the RL approach was better for intracanalicular tumors, the MF was better for tumors smaller than 1.5 cm, and the RS was better for larger tumors.

When comparing MF and RS surgery, with MF being used only for small and intracanalicular tumors and RS for tumors of all size, there was no statistically significant difference in preservation of facial nerve function, despite a difference in percentage (80.7% and 96.5%, respectively).^147^ When comparing TLB and RS surgery, with TLB being used for tumors of variable size and RS for tumors smaller than 3 cm, a total hearing preservation rate of 90% was found, with no statistically significant difference between the techniques.^77^

Although published studies on VS surgery have reported encouraging rates of facial nerve function preservation, specific indications for each approach, such as hearing preservation, tumor size and location, and surgeon experience, make it difficult to assess which is superior. Therefore, in agreement with the Congress of Neurological Surgeons Systematic Review and Evidence-Based Guidelines on Surgical Resection for the Treatment of Patients with Vestibular Schwannomas^193^:

1 There is insufficient evidence to support the superiority of either the MF or the RS approach for facial nerve function preservation in VS surgery when serviceable hearing is present.
2 There is insufficient evidence to support the superiority of either the RS or the TLB approach for facial nerve function preservation in VS surgery when serviceable hearing is not present.
3 There is insufficient evidence to support stating that a multidisciplinary team consisting of a neurosurgeon and a neurotologist provides superior outcomes compared with either subspecialist working alone.

### CI in patients with NF2-related VS

The CI has been recommended as an auditory rehabilitation method for hearing loss associated with sporadic or NF2-related VS, whether resulting from the disease itself or the treatment used.^300^, ^301^, ^302^, ^303^, ^304^ Initially, the CI was only indicated in patients with bilateral hearing loss, but is currently indicated for unilateral hearing loss as well, especially in patients with tinnitus,^127^, ^305^, ^306^ with the goal of reestablishing the benefits of binaural hearing, such as enhanced spatial location and noise discrimination.^300^ There are no standard criteria between different services for the indication of surgery, hearing evaluation, or preoperative prognosis.^300^, ^301^, ^302^

Hearing loss was defined as ≤50% monosyllable recognition at 80 dB by Arnoldner et al.,^303^ as mean tonal thresholds >70 dB by Deep et al.,^307^ and as tonal thresholds >90 dB by Mukherjee et al.^308^ Sorrentino et al.^301^ considered CI indication for patients with moderate-to-severe hearing loss in the contralateral ear and TLB surgery in the ipsilateral ear. The use of electrically eABR has been shown to be effective in the intraoperative assessment of cochlear nerve function after VS resection, assisting in the selection of patients who may benefit from a CI.^306^, ^309^

The CI is considered an option in cases where there is anatomical and functional preservation of the cochlear nerve, since CI results can be superior to those of the Auditory Brainstem Implant (ABI), with less morbidity.^127^, ^309^ Preservation of the cochlear nerve and improved CI response have been reported for tumors of up to 2 cm.^307^, ^310^, ^311^ The CI has been used in “wait and scan” patients with VS who showed growth stability in the last2 to 3 years^310^, ^312^, ^313^; in patients undergoing microsurgery with a hearing preservation technique such as the MF, RLB, or RS that evolves with hearing loss^305^, ^310^ or with the TLB approach,^300^, ^306^ either simultaneously or sequentially; and in patients undergoing radiosurgery or radiotherapy.^114^, ^300^, ^306^ When performing sequential CI, the placement of a dummy electrode in the cochlea is recommended to maintain permeability.^308^, ^314^, ^315^

Iannaconne et al.^310^ recommend that CI be conducted simultaneously to TLB resection of VS, as studies have reported 40% of cochlear ossification within the first year after the surgery. Conversely, Wick et al.^114^ did not find a significant difference between simultaneous or sequential CI in a literature review of 93 patients. Mukherjee et al.^308^ retrospectively evaluated 11 patients and found better results in patients who underwent surgery or observation instead of irradiation.

In a literature review with 86 patients undergoing VS surgery (via the MF, RL, RS, or TLB approach), West et al.^305^ did not find a significant difference in CI results between patients with sporadic or NF2-related VS, with mean discrimination scores from moderate-to-high performance and only a few cases with no response. The authors emphasized the difficulty of comparing different studies since the tests used for audiologic evaluation were different.

In a prospective study, Arnoldner et al.^303^ considered the presence of positive eABR responses during TLB resection of sporadic VS as an indication of cochlear nerve function preservation to allow simultaneous CI. Of 17 patients, 10 received the CI. The authors also observed improved postoperative responses in patients who had residual speech recognition preoperatively, ranging from 20% to 65% in 9 patients after 6 months of surgery. One patient had no response. In a systematic review of 45 studies including 15 patients with sporadic VS,^316^ the mean speech discrimination score improved from 30% to 56.4%, with better results in patients who presented poorer discrimination preoperatively. Tumor location and size, duration of deafness before CI, and type of CI (sequential or concomitant) were not associated with postoperative hearing outcomes.

Deep et al.^307^ retrospectively evaluated 24 patients with NF2 and prior microsurgery, irradiation, or observation who received a CI. Mean patient follow-up was 4 years. The rate of open-set speech understanding was 42%, with better results in tumors of up to 1.5 cm.

Sanna et al.^306^ retrospectively evaluated 41 patients with VSs < 2 cm (33 sporadic and 8 NF2-related) who underwent unilateral TLB resection with simultaneous CI. One year after the surgery, 48.8% of patients still used their CI, with a sentence recognition rate of 60.7%. Those who stopped using had a sentence recognition rate of 15.3%. There was no statistically significant difference between patients with sporadic or NF2-related VS. Better preoperative audiologic test results had a positive correlation with postoperative results, which may indicate better preservation of neural elements, contrary to the study by Bartindale et al.^316^

Longino et al.^313^ retrospectively evaluated 7 patients with observed sporadic VS that showed tumor growth stability. Consonant-nucleus-consonant word scores improved from 6% to 55% and AzBio scores in quiet improved from 9% to 56% 6 months after CI and were maintained at 12 months.

Sorrentino et al.^301^ evaluated 17 patients (8 with sporadic and 9 with NF2-related VS) after 24 months of CI; one of the patients received a bilateral CI. CI was simultaneous in 11 ears and sequential in 7 and occurred between 1 and 3 months after VS resection. The results were better in patients with sporadic VS, worse in those with better contralateral hearing, and preoperative hearing had no effect on the implanted side. The mean word recognition score was 40%.

Regarding tinnitus, West et al.^305^ retrospectively evaluated 22 patients, 21 with sporadic and 1 with NF2-related VS. Nineteen patients underwent tumor resection, 1 underwent radiotherapy, and 2 were only observed. Sixteen cases had single-sided deafness and 1 case had bilateral normal hearing. The Tinnitus Handicap Inventory (THI) was applied in 17 patients pre and postoperatively, with significant score reductions, increases, and maintenance in 76%, 6%, and 18% of them, respectively. Conway et al.^127^ prospectively evaluated 10 patients with sporadic VS who underwent TLB resection with concomitant CI and found a significant reduction in THI scores (from 41.3 to 23.3) after 3 months of surgery. Tian et al.^317^ reviewed 33 patients who received radiotherapy and noted improvement in speech discrimination and reduction in tinnitus in 70% of them after CI.

There is concern regarding the effectiveness of imaging tests in detecting tumor resection, recurrence, or control of residual tumor. However, satisfactory evaluation has been possible. Novel bipolar magnets align with the magnetic field and reduce the chances of CI displacement and pain during the exam. Modifications in CI positioning have allowed MRI to be performed with 1.5-T and 3.0-T machines without causing many artifacts in the IAC region.^318^, ^319^ As for the surgical technique, positioning the receiver 8–9 cm from the EAC at an angle of 90 ° or 160 ° in relation to a line that passes through the nasion (anterior portion of the frontonasal suture) and the EAC allows adequate visualization of the IAC and the CPA.^318^, ^319^

#### Recommendations

XXIX – The CI may be indicated in patients with single-sided deafness and chronic intractable tinnitus due to sporadic VS undergoing surgery or radiotherapy when there is anatomical and functional preservation of the cochlear nerve. In this scenario, the risks and benefits should be considered, as well as the additional care required for postoperative radiological follow-up. Moderate recommendation, moderate quality of evidence.
XXX – Patients with NF2-related VS may receive a CI even if the tumor was not removed. Also, when partially removed (if it is neurologically safe) since it can increase the chances of cochlear nerve function preservation. Moderate recommendation, moderate quality of evidence.
XXXI – Patients with intracochlear or intralabyrinthine VS can receive a CI even if the tumor was not removed. Moderate recommendation, low level of evidence.

### Indications and results of Auditory Brainstem Implant (ABI) in vestibular schwannoma

The ABI is a semi-implantable device designed to restore some hearing function in patients rendered deaf by cochlear or cochlear nerve trauma that makes CI surgery impossible. The most common cause of bilateral impairment of cochlear nerves is NF2. The device is placed directly on the cochlear nuclei in the fourth ventricle, and this procedure may be performed simultaneously to the removal of one of the auditory nerve tumors after the tumor has grown or compromised serviceable hearing.

Similar to the CI, the ABI has an internal and an external component. The internal apparatus is called a receiver-stimulator and, together with an electrode array mounted on a silicone paddle, is surgically implanted. The external apparatus consists of a microphone, speech processor, and transmitter coil. A small microphone on the speech processor captures sound, digitizes it, and sends it to the transmitter coil. The transmitter coil is held over the receiver-stimulator by a magnet, close to the ear.

The implanted receiver sends signals to the electrodes on the silicone paddle that is placed along the surface of the brainstem, allowing direct contact with the cochlear nuclei (which have their own tonotopy). These stimuli produce responses that are interpreted by the brain as sounds, auditory sensations, most of which are not discriminated in detail. One of the main goals of NF2 treatment is to preserve hearing via early diagnosis and treatment of acoustic neuromas.

In patients with NF2, continuous follow-up with MRI is critical; however, the ABI is MRI compatible (at 1.5 T) only with removal of the magnet from the receiver-stimulator. Therefore, during the implantation procedure, the magnet used to couple the transmitter to the implanted receiver must be removed in patients with NF2, and the external transmitter coil must be always attached to the scalp using an adhesive disk. The multichannel ABI is approved for use only in patients who are at least 12 years old and have language competency.^320^

#### Surgical access to the cochlear nuclei

The choice of surgical approach (TLB, RS, or RL) is based on tumor size and the surgeon’s preference. It should guide the placement of the electrode array through the lateral recess of the IV ventricle (foramen of Luschka). The entrance of the recess is marked by the choroid plexus. The lateral recess is also defined by CN VIII, whose root forms its superior wall, and by CN IX, which exits the brain along its inferior edge.

The lateral end of the IV ventricle, the foramen of Luschka, is located between the outputs of the glossopharyngeal and facial nerves. As the floculus is moved away, the surgeon can visualize a depression between the above mentioned CNs, which is the site where the electrode is to be inserted. Usually only one stump of the cochlear nerve can be identified, and it may also be used as a landmark for the lateral recess.

After adequate positioning of the electrode array on the ventral cochlear nucleus, the magnet is removed from the receiver-stimulator and replaced with a nonmagnetic one. The receiver–stimulator is carefully positioned under the temporal periosteum, and the ground electrode is placed in the temporalis muscle for closing.

What makes it possible to find the ventral nucleus of the cochlear nerve without seeing it is the surgeon’s knowledge of anatomical landmarks and a multidisciplinary team with an electrophysiologist and efficient speech therapists. Intraoperative monitoring of EABRs allows proper positioning of the electrode array. Monitoring of CN VII and lower CN IX, X, and XI responses is also performed, as well monitoring of cardiac and vocal fold activity using endotracheal tube-based surface electrodes.

#### ABI results

ABI activation is done after 6–8 weeks of the procedure, in an intensive care unit or operating room, with monitoring of vital signs. Sometimes, specific electrodes may need to be turned off because of distressing nonauditory effects.

Published studies have not found an association between tumor size and ABI results.^321^ ABI placement is just one of the steps toward restoring hearing. After surgery, the patient must attend several sessions with a speech therapist to test and adjust the speech processor programming and to learn to interpret new sounds. This process can take a long time, as the acoustic cues generated by the ABI are different from those of normal hearing.

Medical and audiologic follow-up is usually performed every 3 months in the first year and then annually. Patient counseling should emphasize that sounds can be very off-putting at first but, with time and training, sound quality will slowly improve. Most patients benefit from the ABI in daily life, particularly in combination with lip reading.

ABI results demonstrate greater unpredictability regarding sound detection and closed- and open-set speech recognition and discrimination. As for QoL questionnaires, patients without residual hearing show improved scores. In general, it is safe to say that most patients will experience auditory perception and, especially, an improvement in communication combined with lip reading. However, questions remain about which factors of ABI positioning can influence outcomes.

#### Recommendations

XXXII – Patients with sporadic VS and single-sided deafness may benefit from auditory rehabilitation with Contralateral Routing of Signal (CROS) and Bone-Anchored Devices (BAD). Weak recommendation. Low degree of evidence.
XXXIII – The CI is indicated as initial therapy in patients with NF2 and a positive EABR. Patients with a negative EABR but with anatomical preservation of the cochlear nerve may also benefit from CI before ABI, considering the superiority of the CI in relation to the ABI for NF2 patients and bilateral severe-profound hearing loss. Weak recommendation. Low degree of evidence.

## Conclusion

Decision making in VS treatment has become more challenging. MRI can diagnose increasingly smaller tumors, which has disastrous consequences for the patients and their families. It is important to develop an individualized approach for each case, which highly depends on the experience of each surgical team. We describe below recommendations for different situations according to the evidence described in this Guideline.

### Sporadic VS

XXXIV – Intralabyrinthine or intracochlear tumors – “Wait and Scan Protocol”. Strong recommendation. Moderate degree of evidence.
XXXV – Koos 1 and normal hearing – “Wait and Scan Protocol”. MRI and hearing evaluation every 6 months. Strong recommendation. Moderate degree of evidence.
XXXVI – Koos 1 and ipsilateral anacusis – “Wait and Scan Protocol”. MRI every 6 months. Strong recommendation. Moderate degree of evidence.
XXXVII – Koos 1 – if evidence of VS growth (≥2 mm) exam and/or progressive SNHL. Consider intervention.
  a) Surgery – FM/RL/RS – Moderate recommendation. Low degree of evidence.
  b) Patients with clinical contraindications to surgery – Radiotherapy – Moderate recommendation. Low degree of evidence.
XXXVIII – Koos 2 – “Wait and Scan Protocol” or Intervention (consider age and hearing). Moderate recommendation. Moderate degree of evidence.
XXXIX – Koos 2 and progressive SNHL and/or progressive growth ≥2 mm) – Consider intervention.
  a) Surgery – FM/RL/RS – Moderate recommendation. Low degree of evidence.
  b) Patients with clinical contraindications to surgery. Moderate recommendation. Low degree of evidence.
XL – Koos 3 and normal hearing – Intervention.
  a) Surgery – FM/RL/RS. When the tumor has reached the IAC fundus, even if the patient has serviceable hearing, TLB resection could be indicated because of low probability of hearing preservation. – Moderate recommendation. Low degree of evidence.
  b) Patients with clinical contraindications to surgery. If evidence of tumor growth and/or no serviceable hearing – Radiosurgery. Moderate recommendation. Low degree of evidence.
XLI – Koos 4 – Intervention
  a) Surgery – RS/TLB. When the tumor has reached the IAC fundus, even if the patient has serviceable hearing, TLB resection could be indicated because of low probability of hearing preservation. – Strong recommendation. Low degree of evidence.
  b) Radiosurgery (high risk of mass effect and hydrocephalus). Not recommended.

### Unilateral or bilateral NF2-related VS

The high incidence of newly developed tumors, rapid tumor growth, and early tumor growth, as well as the lack of a cure, make patient management challenging. In most patients, several consecutive therapies must be performed to preserve CN and brainstem function.

XLII – In large bilateral VS with brainstem compression and signs of intracranial hypertension, surgical management should be performed as soon as possible. Strong recommendation. Moderate degree of evidence.
XLIII – If near-total resection is performed to preserve CN VII/VIII function, residual tumors may be treated with SRS. Moderate recommendation, low degree of evidence.
XLIV – “Wait and Scan Protocol” may be considered in residual/recurrent tumors, but each case should be assessed individually. In cases of progressive growth, treatment depends on the patient’s clinical condition, CN function, tumor size, and previous therapies. Weak recommendation, moderate degree of evidence.
XLV – Bevacizumab has had positive effects on hearing preservation and tumor growth in approximately one-third of patients. Moderate recommendation, moderate degree of evidence.
XLVI – Because VS compromises hearing function, auditory rehabilitation is critical for these patients, especially in case of severe-to-profound bilateral SNHL. Strong recommendation, moderate degree of evidence.

## Conflicts of interest

The authors declare no have conflicts of interest.
